# 4-bit adhesion logic enables universal multicellular interface patterning

**DOI:** 10.1038/s41586-022-04944-2

**Published:** 2022-08-10

**Authors:** Honesty Kim, Dominic J. Skinner, David S. Glass, Alexander E. Hamby, Bradey A. R. Stuart, Jörn Dunkel, Ingmar H. Riedel-Kruse

**Affiliations:** 1 Department of Molecular and Cellular Biology, University of Arizona, Tucson, AZ USA; 2 Department of Mathematics, Massachusetts Institute of Technology, Cambridge, MA USA; 3 Department of Molecular Cell Biology, Weizmann Institute of Science, Rehovot, Israel; 4 Department of Applied Mathematics, University of Arizona, Tucson, AZ USA; 5 Department of Biomedical Engineering, University of Arizona, Tucson, AZ USA

**Keywords:** Biomaterials, Synthetic biology, Biophysics

## Abstract

Multicellular systems, from bacterial biofilms to human organs, form interfaces (or boundaries) between different cell collectives to spatially organize versatile functions^1,2^. The evolution of sufficiently descriptive genetic toolkits probably triggered the explosion of complex multicellular life and patterning^3,4^. Synthetic biology aims to engineer multicellular systems for practical applications and to serve as a build-to-understand methodology for natural systems^5–8^. However, our ability to engineer multicellular interface patterns^2,9^ is still very limited, as synthetic cell–cell adhesion toolkits and suitable patterning algorithms are underdeveloped^5,7,10–13^. Here we introduce a synthetic cell–cell adhesin logic with swarming bacteria and establish the precise engineering, predictive modelling and algorithmic programming of multicellular interface patterns. We demonstrate interface generation through a swarming adhesion mechanism, quantitative control over interface geometry and adhesion-mediated analogues of developmental organizers and morphogen fields. Using tiling and four-colour-mapping concepts, we identify algorithms for creating universal target patterns. This synthetic 4-bit adhesion logic advances practical applications such as human-readable molecular diagnostics, spatial fluid control on biological surfaces and programmable self-growing materials^5–8,14^. Notably, a minimal set of just four adhesins represents 4 bits of information that suffice to program universal tessellation patterns, implying a low critical threshold for the evolution and engineering of complex multicellular systems^3,5^.

## Abstract (web summary)

A synthetic cell-cell adhesion logic using swarming E. coli with 4 bits of information is introduced, enabling the programming of interfaces that combine to form universal tessellation patterns over a large scale.

## Main

Bacterial communities can produce multicellular interfaces through cooperation and competition^15,16^. Plants, fungi and animals can establish tissue boundary patterns through developmental programmes using independently evolved yet similar genetic toolkits, organizers and morphogen fields^1,3,4,17–19^. Mathematically, such interface (or boundary) patterns can often be described as tilings or tessellations^20,21^, which elegantly combine generic algorithmic design principles with functional structure and aesthetic appeal, as exemplified by neuronal self-avoidance tilings^22,23^, DNA tiling self-assembly^24^, Islamic architecture^25^ and the Tetris video game^26^. Synthetic biology aims to engineer patterned multicellular systems to facilitate applications including programmable biomaterials, artificial tissues and metabolic consortia, and to provide a build-to-understand methodology for natural systems^5–8^. Despite recent bioengineering advances^5,7^, experimentally feasible algorithms and standardized modules for multicellular interface patterning are limited, in part because the design potential of cell–cell adhesion^5,7,10–12^ is underexplored compared with cell–cell signalling^13,27–30^.

## Multicellular interface patterns

**Fig. 1:**
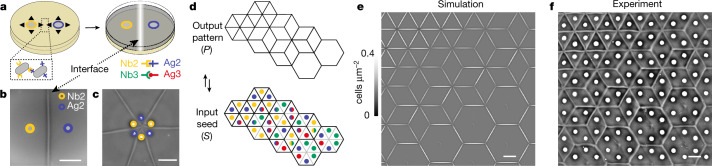
Swarming *E. coli* expressing heterophilic synthetic cell–cell adhesins form programmable interfaces, which enables complex tessellation and tiling patterns at the tissue-level scale^20,21^. **a**, Schematic of the experimental procedure: overnight cultures of swarming *E. coli* seeded on soft agar form macroscopically visible interfaces when expressing complementary pairs from a library of heterophilic synthetic adhesins (Nb2/Ag2 and Nb3/Ag3 corresponding to yellow/blue and green/red, respectively). **b**, A visible interface is formed between two colonies with complementary adhesins (Supplementary Video S1). Scale bar, 5 mm. **c**, Six alternating colonies form a hexagonally symmetric interface pattern. Scale bar, 9 mm. **d**, Observations (**a**–**c**) suggest the potential to engineer more complex 2D target patterns *P* (top), posing the inverse design problem of finding valid seeding patterns *S* of cells (bottom) that have the necessary spatial organization and express suitable adhesins. Seeding positions are colour-coded by strain as in **a**, with mixed-colour half-circles representing homogeneous mixtures of strains. The approach to find the seeding pattern is described in the main text (see also Supplementary Text S2). **e**,**f**, Simulations (**e**) based on the periodically repeating seeding pattern in **d** quantitatively predict the experimentally observed interface patterns (**f**) (Supplementary Videos S2 and S3), realizing an Escher-like^32^ transmutation. Scale bars, 5 mm. Throughout this work, non-linear background subtraction was consistently applied to all non-fluorescent macroscopic images to correct for inhomogeneous illumination and improve visual contrast; this had no impact on image analysis or interpretation (see Methods).

Here we introduce the engineering, modelling and algorithmic programming of multicellular interface patterns by realizing a synthetic cell–cell adhesin logic with swarming bacteria (Fig. 1). We seeded two synthetic adhesin-expressing *Escherichia coli* colonies^10^ a few millimetres apart on soft agar and let them grow, divide and migrate towards each other overnight in pseudo-2D swarms (Fig. 1a, Supplementary Video S1 and Methods). These bacterial-surface-displayed synthetic adhesins were derived from nanobodies (Nb) and their complementary antigens (Ag): Nb2 binds Ag2, Nb3 binds Ag3, but Nb2 does not bind Ag3 etc.^10,31^ (Fig. 1a). We then discovered that a macroscopically visible interface forms between any two strains expressing complementary heterophilic adhesins, such as Nb2 and Ag2 (Fig. 1b). Seeding more colonies generated further interfaces predictably, for example, seeding six alternating Nb2/Ag2 colonies in a circle produced star-shaped interface patterns (Fig. 1c).

This suggests that complex 2D target patterns *P* could be generated by specifying suitable adhesin combinations and seed patterns *S* (Fig. 1d). For example, we asked whether one can program bacterial swarms to generate an Escher-like transmutation^32^ between triangles, cubes and hexagons (Fig. 1d). Indeed, this can be achieved with just four adhesins by solving the corresponding inverse design problem (Fig. 1e,f, Supplementary Fig. S9, Supplementary Texts S1 and S2 and Supplementary Videos S2 and S3; throughout the paper, see Supplementary Figure S10 for accessible colourings). In the following, we systematically investigate the biophysical mechanisms underlying this interface formation to establish a comprehensive adhesion logic framework for the inverse design of arbitrarily tessellated interface patterns^20,21^ (Supplementary Text S2).

## Biophysics of interface formation

**Fig. 2:**
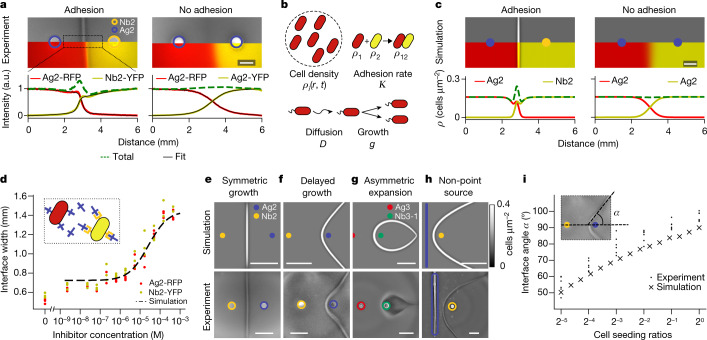
Key interface properties can be rationally controlled in quantitative agreement with a biophysical continuum model. **a**, Composite images of oblique transmitted illumination microscopy and fluorescent microscopy (top) and normalized quantification of fluorescence (bottom) show sharp and gradual interfacial transitions with adhesion (left) and without adhesion (right), respectively (for fit, see equation (1)). Note that colours of fluorophores (RFP, YFP) should not be confused with colour labels (blue, yellow) for two adhesins. Scale bar, 2 mm. **b**, The continuum model incorporates cell density, adhesion (leading to aggregation and immobilization), (active) diffusion and logistic population growth (equation S1). **c**, Model simulations recapitulate experiments and interface profiles from **a** (Supplementary Video S4). Scale bar, 2 mm. **d**, Interface widths can be experimentally tuned by adjusting adhesion avidity (*K*) using an adhesin inhibitor (EPEA^10^). **e**, Symmetric interfaces form when complementary strains have identical growth and motility properties. Scale bars, 5 mm. **f**, Interface angle and position can be tuned by delaying swarming initiation for one of the two colonies (experimentally, by using lower seeding density). Scale bars, 5 mm. **g**, Interface curvature and position can be tuned by using strains with different expansion rates (Supplementary Video S5). Scale bars, 5 mm. **h**, Non-point-source seeding generates interfaces consistent with the linear superposition of many point sources; for example, a line and a point generate the mathematically expected parabolic interface. Scale bars, 5 mm. **i**, Model and experiment agree for interface angle over a wide range of seeding densities (compare to **f**; see also Supplementary Fig. S2).

We first confirmed that swarming *E. coli* cells form macroscopically visible interfaces if and only if complementary adhesins are present in adjacent cell populations (Fig. 2a and Supplementary Fig. S11). We seeded a pair of colonies with complementary adhesins that cytoplasmically express red and yellow fluorescent proteins (Ag2-RFP|Nb2-YFP) (Fig. 2a, left). We fitted a generalized Hill function to the resulting fluorescence profiles (equation (1)) and determined the width of a transition region for adhesive cell pairings to be 500 ± 10 μm (mean ± standard deviation, *n* = 3 replicates throughout if not stated otherwise), which was significantly narrower than 1,290 ± 20 μm (*p* < 0.001, Tukey’s honestly significant difference test) for non-adhesive pairings (Ag2-RFP|Ag2-YFP) (Fig. 2a, left/right). As the interface remains much wider than the size of a single cell (about 2 μm), the narrowing of the transition region suggests that cells are intermixed with incomplete blocking. Furthermore, the total cell density is characteristically increased at the interface, with cell density reductions on either side. Without adhesion, both strains combine to a nearly homogeneous distribution (Fig. 2a, right). Hence, these adhesins mediate a new type of emergent density patterning distinct from previously established mechanisms relying on signalling and motility^29^ or interstrain competition^30^.

To quantitatively capture this interface-forming behaviour and larger-scale patterning, we introduced adhesion into a minimal continuum model of bacterial swarming^30^ (Fig. 2b and Supplementary Text S1). Each strain *i* is described by a spatio-temporal density *ρ*_*i*_(*r*, *t*) (*r* position, *t* time), which grows to saturation *ρ*_max_ at rate *g*_*i*_, spreads with effective diffusion constant *D*_*i*_ and interacts with a complementary adhesive strain *j* to form a new phase *ρ*_*ij*_ through adhesion of strength *K*. In accordance with our previous results^10^, the adhesion is considered irreversible. Using initial conditions of two spatially localized strains, the model recapitulates the outgrowing wavefronts, as well as the characteristic peak and troughs at the interface, which are absent without the adhesins (Fig. 2c and Supplementary Video S4). Matching simulation and experiment yielded biologically reasonable estimates for all model parameters (*D* = 47 ± 8 μm^2^ s^−1^, *g* = 14 ± 6 h^−1^, *K* = 130 ± 50 h^−1^) (Fig. 2c).

This model predicts that tuning seeding conditions enables quantitative control over various geometric interface properties, which we confirmed experimentally (Fig. 2d–i and Supplementary Text S1). Decreasing the adhesion binding rate *K* should widen the interface (Fig. 2d), which we confirmed by adding a small peptide competitive inhibitor (EPEA) against Nb2 (ref. ^10^) to quantitatively titrate adhesin levels; varying inducer concentration produced equivalent results (Supplementary Fig. S12). Delaying the expansion of one colony by varying the relative initial seeding concentrations (Supplementary Fig. S13) should produce interfaces angled and shifted towards the delayed colony (Fig. 2e versus 2f), which we confirmed over a wide range of seeding ratios (Fig. 2i). Differences in expansion rate *D*_1_ versus *D*_2_ should lead to interfaces curved towards the slower-growing colony—even engulfing them (Fig. 2g and Supplementary Video S5), which we confirmed by using a slow-growing Nb3 variant, Nb3-1 (ref. ^10^). The Nb3, Ag3, Ag2 and Nb2 lines all have comparable expansion rates (Supplementary Fig. S14). Non-point-source seeding should behave like a summation of many point sources, which we confirmed by seeding one strain in a line, producing the expected parabola (Fig. 2h and Supplementary Fig. S15). For extensions to the model, see Supplementary Texts S1.6 and S1.7 and Supplementary Video S6.

## Swarming adhesion mechanism

**Fig. 3:**
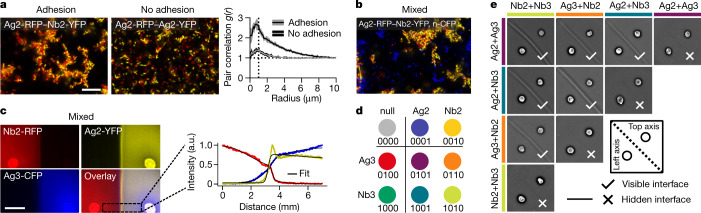
At the microscopic intercellular level, interfaces are formed by means of a ‘swarming adhesion’ mechanism, which enables a combinatorial 4-bit cell–cell adhesion logic to control interface formation. **a**, Confocal images show that adhesive cells form a porous interface with punctate cellular aggregates (left) compared with substantially smaller clusters without adhesins (middle). Multicomponent pair correlation (right) between different cell types in adhesive (black) and non-adhesive (grey) conditions. Solid lines and shaded area are mean and 1 standard deviation, n=9 experiments, dashed lines are a visual guide for *g*(*r*) = 1 and radius = 1 μm. Scale bar, 15 μm. **b**, Three-colour confocal image for interface region includes an extra non-adhesive strain (CFP), which is excluded from the adhesive cell clusters (RFP and YFP). **c**, Epifluorescent images of a three-strain experiment, with non-adhesive (CFP) and adhesive (RFP, YFP) strains (left). Fluorescent profiles of mixed culture experiment (right); colours correspond to the expressed fluorophore, black curve is fit to model as in Fig. 2a. Scale bar, 3 mm. **d**, 4-bit cell–cell adhesion logic: composable set based on two adhesin pairs depicting the nine elements of practical interest, that is, the ‘null’ element without adhesins, four strains with a single adhesin (‘singlets’) and four strains with two adhesins (‘doublets’). Colour-coding corresponds to adhesin identity (singlets as in Fig. 1a); not to be confused with fluorescent colours. (Note that in Fig. 1d, adhesin mixes were represented by two half-circles instead.) **e**, Pairwise combinations of all doublet elements follow expected interface formation logic. Scale bar, 9 mm.

To gain a mechanistic understanding of interface formation at the microscopic level, we imaged the interfaces and surrounding regions using confocal microscopy. We found that cells form an approximately 0.4-mm-thick layer, do not bulge upwards at the interface (within 10-μm measurement resolution) and only fill 20–25% of the available space (Fig. 3a and Supplementary Figs. S16–S24). Individual cells invade the opposing region, explaining the observed transition region of many cell widths (Fig. 2a and Supplementary Fig. S18). In this transition region, complementary adhesive strains form clusters, whereas non-adhesive control strains do not, as quantified by the greater first peak in the two interstrain pair-correlation functions for the adhesive strains (Fig. 3a). This correlation function also had a larger characteristic width, as quantified by the variance of an exponentially modified Gaussian fit (5.24 ± 1.09 μm^2^ and 0.91 ± 0.38 μm^2^ for the adhesive and non-adhesive pairs, respectively, *p* < 0.001 independent *t*-test, *n* = 9 replicates, Supplementary Fig. S19). Confocal stacks show that the interface clusters form structures embedded in the agar with features that are at least tens of microns in size and seem highly interconnected (Supplementary Figs. S20–S22). Time-lapse microscopy shows that cells migrate as a collective and become immobilized at the interface (Supplementary Video S7). Immobile cell clusters then act as sinks for swarming adhesive cells at the interface. Further discussion and quantification including unaveraged pair-correlation plots is provided in Supplementary Fig. S23. Adhesive cluster size and mean free path length are also increased compared with the control (Supplementary Fig. S24). These data recapitulate and explain the sharp versus gradual transitions seen in wide-field images of the adhesive versus non-adhesive pairs, respectively (Fig. 2a). Hence, we identified a ‘swarming adhesion’ mechanism in which long-range swarming and short-range adhesion generate interfaces that are many cells wide and have low cell densities, which is distinct from previously established tissue segmenting and interface-generating mechanisms, for example, based on differential adhesion or jamming^1,23^.

## Adhesion logic and interface composability

The large width and sparseness of these interfaces suggested that they could discriminate between cells with complementary versus non-complementary adhesins. To test this hypothesis, we performed interface-formation experiments with complementary adhesins as before, but now mixed one of the complementary strains with a third non-binding orthogonal strain expressing Cerulean (CFP). Confocal imaging showed that an orthogonal strain (n-CFP, in which n denotes a null adhesin control) does not bind to adhesive clusters (Fig. 3b), with a characteristic pair-correlation width of 8.46 ± 2.71 μm^2^ for the adhesive case, and no meaningful fit was possible for the non-adhesive cases (Supplementary Figs. S19 and S23). Epifluorescent imaging similarly showed that a non-complementary strain (Ag3-CFP) infiltrates further than either of the complementary strains (Fig. 3c). Control experiments with various fluorophore and adhesin combinations indicate that these results are not due to potential differences in migration rates between strains (Supplementary Fig. S25). We conclude that these interfaces are semipermeable by selectively filtering complementary cells while allowing non-complementary cells to pass.

## 4-bit adhesion logic

The observation that introducing a third non-complementary adhesive strain does not interfere with interface formation (Fig. 3c) suggests that multistrain mixtures in general could show composability^7^. A library of two adhesin pairs can be combined into 16 possible ‘elements’ at each seeding position, corresponding to 4 bits of information, and in which nine elements are of practical use here (Fig. 3d and Supplementary Text S2): a ‘null’ element Σ_0_ = {*n*} without any adhesin, a subset of four ‘singlet’ elements Σ_1_ = {Nb2, Ag2, Nb3, Ag3} and a subset of four ‘doublet’ elements Σ_2_ = {Nb2 + Nb3, Nb2 + Ag3, Nb3 + Ag2, Ag2 + Ag3}. Any other combination is not of practical interest here owing to self-interaction but might have applications in future patterning processes. We prepared these four doublet mixtures and performed interface-formation experiments as before. As expected, we found that a visible interface between two doublet elements is formed if and only if they are different (Fig. 3e). We also introduce the term ‘hidden’ interfaces for cases in which no visible interfaces form, which will become conceptually useful later in the paper. We also experimentally tested various pairings of singlet versus doublet elements, which all followed the expected interface-formation logic (Supplementary Fig. S25). Thus, these orthogonal adhesins and the corresponding composable sets and subsets of strains^10^ (Fig. 3d) provide a cell–cell adhesion logic for generating visible and hidden interfaces (Fig. 3e).

## Universal interface and tessellation patterns

**Fig. 4:**
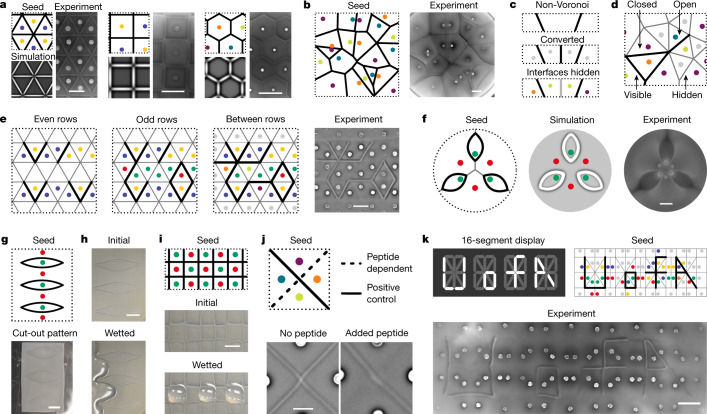
General interface patterning can be achieved with just four adhesins, providing an engineering platform for applications such as smart biomaterials and human-readable molecular diagnostic devices. **a**, Generating regular triangular, square and hexagonal tilings requires one to two adhesin pairs. Scale bars, 3 mm (left) and 9 mm (middle and right). **b**, Generating Voronoi tessellation with four doublet elements is guaranteed by the four-colour theorem (Supplementary Video S8). Scale bar, 9 mm. **c**, Schematic of how a non-Voronoi tessellation can be realized by solving for the associated Voronoi pattern and hiding the added interfaces. **d**, Definition of closed, open, visible and hidden interfaces. General open interface patterns can be realized using only the four doublet elements and the null element. **e**, A three-step algorithm for sequentially identifying seed configurations on regular triangular lattices enables arbitrary interface patterning using only two adhesin pairs (Supplementary Video S9). Scale bar, 9 mm. **f**, Complex curved interface patterns generated with differential expansion rates and seeding densities (green indicates the Nb3-1 strain). Scale bar, 9 mm. **g**, Thin sheets with interface patterns can be cut out and handled, shown here floating in a PBS bath. Scale bar, 1 cm. **h**, Surface wettability of biomaterials can be patterned owing to differential hydrophilicity of interface patterns. PBS added to an interface pattern shows that the liquid surface closely tracks the patterned interface (Supplementary Fig. S29). Scale bar, 1 cm. **i**, Liquid droplets can be captured in regularly tiled squares (Supplementary Fig. S29). Scale bar, 5 mm. **j**, Demonstration of an environmentally dependent patterning and molecular diagnostic application in which a human-readable indicator (‘\’ or ‘X’) is formed, depending on whether a molecular inhibitor against the Nb2 adhesin (EPEA^10^, Fig. 2d) is present or not. Scale bar, 2 mm. **k**, Implementation of the common 16-segment digital display, programmed to write ‘U of A’, demonstrates complex combinatorial patterning with human-readable output (Supplementary Video S8). Colour codes defined in Fig. 3d; for continuum simulations of **b** and **j**, see Supplementary Fig. S27. Scale bar, 9 mm.

Equipped with this combinatorial cell–cell adhesion logic, we can tackle the inverse design problem of finding suitable seed conditions *S* to achieve a desired target pattern *P* (Fig. 1d). Conceptually, this inverse design problem is closely related to long-standing patterning questions in developmental biology, as initial seeding conditions and swarming cells represent adhesion-based bacterial analogues to the well-established concepts of developmental ‘organizers’ and morphogen ‘fields’, respectively^1–3,17,18^. Central questions are: what is the design space of programmable patterns *P*? How many unique adhesins are minimally required to program *P*? What are efficient algorithms for identifying the necessary adhesin combinations and seeding positions *S*? Using this adhesion logic platform, we found that all three regular periodic tilings^20^ can be created by seeding cells at the tile circumcentres, in which triangular and square tilings require just one adhesin pair from the singlet set, whereas hexagonal tilings require two adhesin pairs combined into doublets (Fig. 4a and Supplementary Fig. S26). Moreover, different seeds can generate the same pattern (Supplementary Text S2).

We also found that any arbitrary tessellation and straight-interface pattern in a 2D plane can be generated with just four adhesins (two adhesin pairs), representing 4 bits (Fig. 3d and Supplementary Text S2.3). To outline the necessary algorithmic procedure, we first note that the interface pattern generated from growth-matched swarming strains seeded at different points is equivalent to the Voronoi tessellation of the seeded points^21,33^. The four-colour theorem^34^ guarantees that one can seed four doublets (Fig. 3d,e) such that adjacent tiles always contain a different doublet, ensuring that all interfaces of the Voronoi tessellation will form (Fig. 4b, Supplementary Fig. S27 and Video S8). If the desired interface pattern does not correspond to a Voronoi tessellation, we can use algorithms that solve the generalized inverse Voronoi problem^21^, which guarantees that any interface pattern can be mapped onto a Voronoi tessellation and provides the necessary seeding positions. This will typically require introducing further interfaces and subtiles. However, by seeding all subtiles within an original tile with the same doublet, all further interfaces within a subtile become ‘hidden’ (Figs. 3e and 4c), and the four-colour theorem applies as before. More generally, any interface pattern can be generated even when one or both ends of some interfaces are not connected to another visible interface (Fig. 4d). The ends of such ‘open’ interfaces can always be isolated by also using the null element in addition to the four doublets. This most general case can then be solved as before (Fig. 4d). These algorithms thus provide a universal design framework for generating an arbitrary pattern *P* by determining the sufficient seeding conditions *S* while using at most four adhesins.

The above algorithms can be readily generalized to implement extra constraints, as commonly encountered in many natural and engineered systems. As an example, we considered the task of generating arbitrary interface patterns on a triangular lattice on which seeding positions are restricted to the centre of each triangle (Fig. 4e). We then identified an efficient algorithm that sequentially assigns adhesins to first generate all non-horizontal interfaces (even and odd rows) and subsequently all horizontal interfaces (between rows) (Fig. 4e, Supplementary Fig. S8, Supplementary Text S2 and Supplementary Video S9). As before, this algorithm only requires four adhesins. An initial investigation into other lattice types suggests trade-offs between seeding restrictions, number of available adhesins and achievable pattern complexity, which then pose a range of new and interesting constrained tessellation problems, especially with regards to the selective formation or omittance of interfaces^20,34^ (Supplementary Fig. S28 and Supplementary Text S2). The patterning space can also be extended to include curved interfaces; for example, using expansion rate differences (Fig. 2g,h) enables threefold floral arrangements (Fig. 4f) that differ strikingly from the sixfold ray pattern in Fig. 1c despite identical seeding positions. The continuum model (Figs. 2b and 4f) allows for cost-efficient quantitative pattern exploration before experimental implementation (Supplementary Text S2.2).

## Applications of self-growing bacterial materials

To explore the potential application space of this bacterial adhesion logic, we sought to spatially pattern the properties of such self-growing bacterial materials. Patterned agar sheets that are approximately 2-mm-thin can be cut out (Fig. 4g and Supplementary Fig. S21), setting the basis for free-standing biomaterials^5^. The wettability of the surface can be spatially controlled, that is, the flow of 1× phosphate-buffered saline (PBS) is hindered at visible Nb3|Ag3 interfaces (Fig. 4h, Supplementary Fig. S29 and Supplementary Video S10), whereas it is not hindered for hidden Nb2|Ag3 or Nb3|Ag2 interfaces (Supplementary Fig. S29). This phenomenon could be used to localize liquid droplets (Fig. 4i and Supplementary Fig. S29) and support open-surface microfluidics^14^. The control over interface width using antagonistic antigens (Fig. 2d) suggests molecular diagnostics that translate molecular concentrations into switchable visual interface patterns (Fig. 4j). Such diagnostics could be manufactured without the need for protein purification and should generalize to any molecule against which a nanobody can be formed (Supplementary Text S3). Other applications include human-readable digital displays^35^ (Fig. 4k, Supplementary Fig. S27 and Supplementary Video S8), precision biofiltering^36^ on the basis of selective interfaces (Fig. 3b,c) and build-to-understand approaches for synthetic microbial communities^6–8^.

## Discussion

The introduced cell–cell adhesion logic and algorithms provide a versatile platform for engineering and investigating multicellular patterning, and may also be applicable to non-bacterial systems, such as active particles or mammalian tissues. In engineered systems, more complex patterning logic could be added; for example, sequentially established interfaces can serve as organizers in hierarchical patterning processes^2^ and conditional patterning can be based on environmental or system-intrinsic inhibitors (Figs. 2d and 4j). In natural systems, a differential cadherin adhesion code coordinates animal morphogenesis^37^, and bacteria, amoebae and fungi use sets of polymorphic adhesins in combinatorial ways for kin discrimination and spatial multicellular patterning^15,19,38^. Notably, the ‘swarming adhesion’ mechanism uncovered here is distinct from established chemical-based and adhesion-based mechanisms^1,17,18,37^, and we speculate that natural microbial communities use related mechanisms to distinguish between kin and foe to protect against invasion^15,39,40^. Our results also provide guidance for non-adhesion-based patterning^13,18^ or for hybrid mechanisms in which adhesins are coordinated by combinatorial juxtacrine signalling such as heterophilic Eph–ephrin interactions^41^. Finally, the fact that a combinatorial adhesion logic with just four adhesins can generate universal interface patterns in a plane establishes a low critical threshold for the evolution and engineering of complex multicellular systems^3,5^.

## Methods

### Plasmids and strains

Plasmids were transformed into chemically competent cells following standard protocol^10^. Plasmids were sourced from our earlier work^10^ and are deposited in Addgene (Supplementary Table S1). To better match the metabolic load between cells, all strains used here expressed at most a single adhesin. However, it is possible to use strains expressing several adhesins. For example, instead of mixing two cell types expressing Ag2 and Ag3, one could use one strain expressing both adhesins^10^. Doing so produces the expected logical interactions that dictate interface formation but with greater variance in growth rates.

### Cell culture

The MG1655 *E. coli* strain obtained from E. Coli Genetic Stock Center (CGSC #6300) was used for all experiments in this study. For overnight growth, cells were shaken at 37 °C and 300 rpm with antibiotics in Luria broth (LB) media. No ATc was added for overnight growth. Cell stocks were stored in glycerol at −80 °C. For experiments, single colonies were inoculated from agar plates containing antibiotics streaked out from frozen stocks.

### Soft agar

To prepare soft agar gels, 20 g l^−1^ of LB broth powder (Affymetrix #75852) and 0.225% w/v Bacto Agar (BD 214050) was added to distilled water. This mixture was autoclaved and allowed to cool to 50 °C before adding antibiotics or ATc inducer as needed. This mixture was then pipetted into Petri dishes, at 10 ml for 10-cm dishes, 5 ml for 60-mm dishes, 2 ml for 35-mm dishes and six-well plates, and 25 ml for 15-cm dishes. The plates were covered and allowed to cool at room temperature for at least 2 h and used on the day of preparation. For confocal imaging, the soft agar percentage was increased to 0.25% w/v for greater imaging stability.

To seed cells for experiments, 1 μl of overnight culture was pipetted onto the surface of the gel without puncturing the surface using low-adhesion tips (VWR 89174-520). For interface-formation experiments, a multichannel pipette was used to ensure consistent 9-mm spacing between the colonies. For experiments needing greater patterning complexity, stencils were cut out of 4.5-mm acrylic sheets (TAP Plastics) using a laser cutter (Dremel LC40). The stencils were designed so that the pipette tips were just above the surface of the agar by using shims and varying the radii of laser-cut holes.

Soft agar plates were placed in a 37 °C incubator right side up, with the lid on and no circulating air. Cells were typically grown for 18 h on soft agar before imaging. Note that *E. coli* transitions to a swarming phenotype in swarming agar, resulting in greater expansion compared with the agar concentrations most commonly used in labs for various purposes. When specified in the text, cells were incubated at room temperature for 48 h, with all other conditions kept constant.

### Small peptide inhibitors

Small peptide inhibitor EPEA and the scrambled PEAE were synthesized by GenScript^10^. Frozen aliquots were stored at −80 °C. To create gels with a specific concentration of inhibitor, aliquots of peptide were first thawed and diluted in distilled water to a 10× working concentration. Soft agar gel solutions were prepared at 1.1× concentration to account for the liquid volume in the 10× peptide solution and kept warm in a 50 °C bath before pouring.

### Titrating cell seeding concentration

For experiments needing variable cell concentrations such as the delayed growth experiment, cells were grown in a shaking incubator to stationary phase overnight and diluted in LB media to the desired concentration immediately before seeding.

### Mixed cell populations

For experiments that required seeding mixed populations of different types, cells were first separately grown in a shaking incubator to stationary phase. Immediately before seeding, cells were pipetted from the stationary cultures and mixed in polymerase chain reaction tubes.

### Imaging

#### Fluorescence stereomicroscopy

Fluorescence stereomicroscopy images were obtained using a Leica M205 fluorescent microscope, with a Planapo 2.0× objective (10450030, Leica Biosystems) and DSR (10447412, Leica Biosystems), YFP (10447410, Leica Biosystems) and CFP (10447409, Leica Biosystems) filter sets. Oblique illumination was used for non-fluorescent channels.

#### Confocal images

Confocal images were captured using an inverted Zeiss LSM700 using 405-nm, 488-nm and 555-nm laser lines and a 63× 1.4-NA (0.19 mm FWD) Plan-Apochromat objective (44 07 62, Carl Zeiss AG). Soft agar plates were allowed to equilibrate to room temperature for at least an hour before imaging. Immediately before imaging, a no. 1.5 glass coverslip was carefully placed on the surface of the agar. The soft agar plates were then mounted upside down using a custom laser-cut stage. Images in a tiled set were taken within a few minutes, owing to viscoelastic creep over time. Confocal stacks were acquired at 2-μm slices up to a depth of approximately 20 μm, at which point soft agar undergoes displacement. Furthermore, low-viscosity immersion oil (Resolve M2000, Epredia) was used to minimize the effects of drag on agar as the stage moved around.

#### Epifluorescent wide-field time lapses

Epifluorescent wide-field time lapses were captured using an inverted Zeiss LSM700, with a 20× Plan-Apochromat objective (440640-9903, Carl Zeiss AG), a 1.4-MP CCD monochrome camera (Zeiss AxioCam MRm, Carl Zeiss AG) and a light-emitting diode (LED) lamp (X-Cite XYLIS, Excelitas Technologies Corp.). Plates were mounted as described above and frames were captured every 2 min.

#### Macroscopic images

Macroscopic images were captured using a DSLR camera (D5600, Nikon). Samples were illuminated using oblique illumination from LED ring lights. To correct for the uneven brightness inherent in oblique illumination, a Gaussian blur filter was applied to a copy of the image and this image was then subtracted from the original image using ImageJ^42^. Subsequently, image contrast was adjusted using ImageJ. These corrections were applied in an unbiased manner solely for the purpose of enhancing visually clarity, and no quantifications were made on these images. Specifically, the following panels in this paper were processed this way: Figs. 1b,c,d, 2a,e,f,g,h, 3e and 4a,b,e,f,g,h.

#### Time-lapse macroscopic images

Time-lapse macroscopic images were captured using a Raspberry Pi Camera V2 controlled by a Raspberry Pi Zero. The sample was placed in a small humidified chamber and an LED ring light was used for intermittent illumination. A custom Python script was written to control the Raspberry Pi Camera V2 and ring light activation. Frames were captured every 5 min.

### Measuring interface profiles

To measure the fluorescence profile along an interface, the fluorescence intensity was measured in raw images along a 6-mm line aligned with the axis connecting the initial seeding points. The fluorescence intensity was binned across a width approximately 1.5 mm thick. These profiles were fit using equation (1).

### Measuring interface angles

To measure the interface angles between seeds, the interface was first manually traced using ImageJ. The angle between each leg of the interface and the line connecting the two starting seeding points was calculated using linear regression in Python.

### Image processing for confocal images

Confocal images were cropped to remove smearing caused by the viscoelastic response of the soft agar to stage motion during imaging. The cropped region was always the top 128 × 2,048 pixels, in an image of 2,048 × 2,048 pixels.

For pair-correlation analysis, confocal images were segmented using DeepCell^43^. Cell positions were defined by the centroids of segmentation results. To calculate the mixed-species pair-correlation function, a custom Python script was used. Briefly, for each cell, the script counted the number of cells in a ring *r* + *dr* away. This count was normalized to the area of the ring, as well as the overall cell density. Area corrections were made for rings cut off by the image edges.

### Fitting interface profiles and calculating interface widths

The transition profiles measured using fluorescent microscopy (such as in the graphs at the bottom of Fig. 2a,c, which were measured from the bottom half of the composite images on top) were fitted using the following heuristic equation:

1 $$f(x)=z/(1+{({k}_{a}/x)}^{n})+y+a{{\rm{e}}}^{(-{x}^{2}/b)}$$

Here *n* is the Hill coefficient, *k*_*a*_ is the *x*-position at half the maximum value along the transition and *z* weights the contribution of the Hill function. The variable *a* weights the contribution of the Gaussian distribution and *b* is the standard deviation of the distribution. *y* is a vertical offset for the overall curve.

The rationale for fitting the profiles to this equation is as follows: equation (1) is a sum of a Hill function and a Gaussian. The Hill function is primarily responsible for fitting the transition region, whereas the Gaussian compensates for any slope in the plateau region of the measured curve.

The width of the transition *w* is then defined as the distance between the two points of maximum curvature *κ*, calculated using:

2 $$\kappa =\frac{| {f}^{{\prime\prime} }(x)| }{{(1+{f}^{{\prime} }{(x)}^{2})}^{\frac{3}{2}}}$$

### Free-standing patterned sheets

To produce a free-standing patterned material, patterned agar was covered with 10 ml 1× PBS before a scalpel was used to cut out a rectangular section. For smaller sections, gentle agitation of the PBS by shaking allowed the cut-out patterned sheet to lift off from the Petri dish. For larger sheets, a thin object such as a scalpel or coverslip was slid under the agar to separate it from the Petri dish. Alternatively, a glass slide or coverslip was placed in the bottom of the Petri dish before pouring the agar. In this case, covering with PBS and using a scalpel to cut along the edge of the glass was sufficient for the patterned sheet to lift off without any further agitation or manipulation.

Sheets up to a few centimetres in length could be transferred between dishes by pouring the PBS, and sheets up to a centimetre in length could be lifted out of the PBS on the edge of a coverslip, scalpel or tweezer.

### Patterned wetting

For wetting experiments, strains were seeded and grown on soft agar at room temperature for 48 h to produce strong interfaces. Then, 1× PBS was poured at one end of the plate and allowed to flow. The shape of the PBS front demonstrates the effect of bacterial patterning on surface wetting. For droplet capture in square patterns, 1× PBS was gradually added to the square using a pipette.

## Online content

Any methods, additional references, Nature Research reporting summaries, source data, extended data, supplementary information, acknowledgements, peer review information; details of author contributions and competing interests; and statements of data and code availability are available at 10.1038/s41586-022-04944-2.

## Supplementary information

Supplementary Materials This file contains Supplementary Table 1; Supplementary Texts 1–4; legends for Supplementary Videos and Supplementary References.

Supplementary Video 1

Supplementary Video 2

Supplementary Video 3

Supplementary Video 4

Supplementary Video 5

Supplementary Video 6

Supplementary Video 7

Supplementary Video 8

Supplementary Video 9

Supplementary Video 10

## Data Availability

All analysed data are available in the manuscript or the supplementary materials. Further raw data supporting the main figures are deposited at 10.7910/DVN/RUMTIV. All other data available on request.

## Code availability

All analysis and modelling scripts are available from GitHub (https://github.com/hirklab/AdhesinLogicProject/).
