# Supplementary material for: 4-bit adhesion logic enables universal multicellular interface patterning
Source: Nature. 2022 Aug 10;608(7922):324–9. doi: 10.1038/s41586-022-04944-2 (PMC9365691; doi:10.1038/s41586-022-04944-2)
Supplement: Supplementary file 1 — This file contains Supplementary Table 1; Supplementary Texts 1–4; legends for Supplementary Videos and Supplementary References. [file 41586_2022_4944_MOESM1_ESM.pdf]

nature portfolio

https://doi.org/10.1038/s41586-022-04944-2

Supplementary information

4-bit adhesion logic enables universal multicellular interface patterning

In the format provided by the
authors and unedited

Supplementary Materials (Kim et al., Nature, 2022)

Supplementary Table S1: Strains and Plasmids

Supplementary Texts S1-S4 (incl. Figs. S1 - S29)

Text S1: Modeling and Simulations (incl. Figs. S1 - S5)

Text S2: Mathematical and Algorithmic Aspects of Interface Pattern Formation (incl. Figs. S6 - S8)

Text S3: Practical Considerations for Applying Adhesin Logic as a Biosensor

Text S4: Additional Experiments, Simulations, Data, and Analysis (incl. Figs. S9 - S29)

Supplementary References

Supplementary Videos S1 - S10 (mp4 format)

Video S1: Timelapse corresponding to figure 1c showing colonies growing towards each other and forming distinct interfaces with complementary adhesins Nb2 and Ag2 (top) and not forming an interface with non-complementary adhesins Ag2 and Ag2 (bottom) (Scale bar is 5mm).

Video S2: Timelapse of transmutation pattern formation in figure 1f; the six larger radially oriented stripes are an imaging artifact caused by agar surface reflecting the imaging setup.

Video S3: Simulation of transmutation pattern formation in Video S2 and figure 1e (Scale bar is 5mm).

Video S4: 2D simulation results with adhesion (top left) and without adhesion (bottom left) corresponding to experiments in Video S1 and Fig. 2a (Scale bar is 5mm). 1D simulation results (cross section of 2D simulation along symmetry line) showing evolution of density profile including the characteristic peak and troughs at the center line with adhesion (top right), and without adhesion (bottom right) (see Fig. 2a)

Video S5: 2D simulations capturing both delayed growth and differential expansion rate (see Fig. 2g) (Scale bar is 5mm).

Video S6: Simulation of multi-swarm wave extension model (see Fig. S4).

Video S7: Epifluorescence timelapse of interface region of migrating swarms at cellular resolution: With adhesion (left; Nb2-YFP cell lines colored in green, Ag2-RFP cell lines colored in red) the immotile clusters of cells are seen in yellow, which grows over time; without adhesion (right, null-YFP cell lines colored in green, Ag2-RFP cell lines colored in red) cells do not form yellow clusters, indicating a lack of adhesion (the global drift is due to relaxation of soft agar).

Video S8: Simulation of arbitrary Voronoi patterning (top, see Fig. 4b), and simulation of 16-segment display patterning (bottom, see Fig. 4k) (Scale bar is 5mm).

Video S9: Timelapse of figure 4e showing algorithmic pattern formation.

Video S10: Experiments showing wettability effects of Nb3|Ag3 interfaces that are not observed for Nb2|Ag3 or Nb3|Ag2 controls (see Fig. S29); replay in real-time.

Table S1: Strains and Plasmids

Name	Plasmids	Description	Addgene #
Ag1	pDSG358 (pSB3K3_TetR_pTet_Neae2v1_A3_Akt3PH)	Akt	115599
Nb1	pDSG372 (pSB3K3_TetR_pTet_Neae2v1_N3-1_antiAkt3PH-3AKH13)	Anti-Akt	115602
Ag2	pDSG318 (pSB3K3_TetR_pTet_Neae2v1_EPEA)	EPEA	115595
Nb2	pDSG320 (pSB3K3_TetR_pTet_Neae2v1_antiEPEA)	Anti-EPEA	115597
Ag3	pDSG319 (pSB3K3_TetR_pTet_Neae2v1_P53TA)	P53	115596
Nb3-1	pDSG398 (pSB3K3_TetR_pTet_Neae2v1_N8-1_antiP53TA-R4P43)	Anti-P53-1	115604
Nb3	pDSG410 (pSB3K3_TetR_pTet_Neae2v1_N8-13_antiP53TA-R3P91)	Anti-P53-13	183902
n	pDSG323 (pSB3K3_TetR_pTet_Neae2v1_Null)	null	115594
Ag2-RFP	pDSG318 (pSB3K3_TetR_pTet_Neae2v1_EPEA)<br>pDSG8 (pSB1C3_mCherry)	EPEA<br>mCherry	115595
Nb2-RFP	pDSG320 (pSB3K3_TetR_pTet_Neae2v1_antiEPEA)<br>pDSG8 (pSB1C3_mCherry)	Anti-EPEA<br>mCherry	115597
Ag3-RFP	pDSG319 (pSB3K3_TetR_pTet_Neae2v1_P53TA)<br>pDSG8 (pSB1C3_mCherry)	P53<br>mCherry	115596
Nb3-1-RFP	pDSG398 (pSB3K3_TetR_pTet_Neae2v1_N8-1_antiP53TA-R4P43)<br>pDSG8 (pSB1C3_mCherry)	Anti-P53-1<br>mCherry	115604
Nb3-RFP	pDSG410 (pSB3K3_TetR_pTet_Neae2v1_N8-13_antiP53TA-R3P91)<br>pDSG8 (pSB1C3_mCherry)	Anti-P53-13<br>mCherry	183902
null-RFP	pDSG323 (pSB3K3_TetR_pTet_Neae2v1_Null)<br>pDSG8 (pSB1C3_mCherry)	null<br>mCherry	115594
Ag2-YFP	pDSG318 (pSB3K3_TetR_pTet_Neae2v1_EPEA)<br>pDSG7 (pSB1C3_Venus)	EPEA,<br>Venus	115595
Nb2-YFP	pDSG320 (pSB3K3_TetR_pTet_Neae2v1_antiEPEA)<br>pDSG7 (pSB1C3_Venus)	Anti-EPEA<br>Venus	115597
Ag3-YFP	pDSG319 (pSB3K3_TetR_pTet_Neae2v1_P53TA)<br>pDSG7 (pSB1C3_Venus)	P53<br>Venus	115596
Nb3-1-YFP	pDSG398 (pSB3K3_TetR_pTet_Neae2v1_N8-1_antiP53TA-R4P43)<br>pDSG7 (pSB1C3_Venus)	Anti-P53-1<br>Venus	115604
Nb3-YFP	pDSG410 (pSB3K3_TetR_pTet_Neae2v1_N8-13_antiP53TA-R3P91)<br>pDSG7 (pSB1C3_Venus)	Anti-P53-13<br>Venus	183902
null-YFP	pDSG323 (pSB3K3_TetR_pTet_Neae2v1_Null)<br>pDSG7 (pSB1C3_Venus)	null<br>Venus	115594
Ag2-CFP	pDSG318 (pSB3K3_TetR_pTet_Neae2v1_EPEA)<br>pDSG6 (pSB1C3_Cerulean)	EPEA<br>Cerulean	115595
Nb2-CFP	pDSG320 (pSB3K3_TetR_pTet_Neae2v1_antiEPEA)<br>pDSG6 (pSB1C3_Cerulean)	Anti-EPEA<br>Cerulean	115597
Ag3-CFP	pDSG319 (pSB3K3_TetR_pTet_Neae2v1_P53TA)<br>pDSG6 (pSB1C3_Cerulean)	P53<br>Cerulean	115596
Nb3-1-CFP	pDSG398 (pSB3K3_TetR_pTet_Neae2v1_N8-1_antiP53TA-R4P43)<br>pDSG6 (pSB1C3_Cerulean)	Anti-P53-1<br>Cerulean	115604
Nb3-CFP	pDSG410 (pSB3K3_TetR_pTet_Neae2v1_N8-13_antiP53TA-R3P91)<br>pDSG6 (pSB1C3_Cerulean)	Anti-P53-13<br>Cerulean	183902
null-CFP	pDSG323 (pSB3K3_TetR_pTet_Neae2v1_Null)<br>pDSG6 (pSB1C3_Cerulean)	null<br>Cerulean	115594

Table S1: Strains and plasmids used in this study. Information on the fluorescent constructs (pDSG6, pDSG7, pDSG8) is provided on GenBank (MH492385, MH492386, MH492388); see also reference [1].

1 Text S1: Modeling and Simulations

1.1 Minimal continuum model

We introduce a minimal continuum model that captures the following key physical effects. (1) The local bacterial density increases over time due to growth, and where the growth rate is limited by a carrying capacity. (2) The edge of the swarm grows and expands into regions that have not yet been colonized via chemotaxis [2, 3]. (3) When cells with complementary adhesin pairs interact, they form non-motile clusters and do not subsequently contribute to the dynamics. (4) The simulations are restricted to 2D as the primary mode of *E. coli* migration is parallel to the planar surface of the gel.

To construct such a model for two species, we track the fields, ρ_1, ρ_2, representing the number density of unbound cells, as well as the field ρ_{12}, representing the density of bound clusters. As the cells are primarily confined to a thin layer on the surface, we take two dimensional density fields, representing the number of cells per unit area. Motile cells perform chemotaxis and move in a direction determined by the gradient of the nutrient concentration, and to fully capture these chemotactic dynamics, additional equations for the concentration field are needed [2]. However, to construct a minimal model of the expansion, we only use diffusion and growth terms, resulting in a Fisher-Kolmogorov like equation which admits propagating fronts [4]. The diffusion term results in a net mass flux from high density to low density regions, whilst the growth term models cells growing exponentially at a rate determined by the nutrient concentration, which we approximate as an instantaneous function of the total cell density. Finally, cells with complementary adhesin pairs bind with an effective rate K and are subsequently immobile. Altogether,

$$\begin{aligned}
\frac{\partial \rho_1}{\partial t} &= g_1\rho_1 c(\bar{\rho}) + D_1\nabla^2\rho_1 - \tilde{K}\rho_1\rho_2,\\
\frac{\partial \rho_2}{\partial t} &= g_2\rho_2 c(\bar{\rho}) + D_2\nabla^2\rho_2 - \tilde{K}\rho_1\rho_2,\\
\frac{\partial \rho_{12}}{\partial t} &= 2\tilde{K}\rho_1\rho_2,\\
c(\bar{\rho}) &= 1 - \bar{\rho}/\rho_{max} = 1 - (\rho_1 + \rho_2 + \rho_{12})/\rho_{max},
\end{aligned} \tag{S1}$$

where g_1, and g_2 are growth rate constants, D_1, D_2 are diffusion constants, ρ_{max} is the carrying capacity for total growth, $\bar{\rho}$ is the total cell density, ρ_1 and ρ_2 are the densities of the two mobile cell populations, ρ_{12} is the density of immobile cells, $\tilde{K} = K/\rho_{max}$, and c is our approximation of the instantaneous nutrient field concentration, allowing for logistic growth until $\bar{\rho} = \rho_{max}$.

1.2 Quantitative model parameter fitting from experiments

We determine these parameters directly from experimental data. Based on our confocal data (Figs. 3a, c, S16), the maximum density achieved during the experiments is $\rho_{max} = 0.155 \pm 0.012\text{cells}/\mu\text{m}^2$, which we note is far from space-filling. We also measure that colonies expand at a rate of $v = 3.04 \pm 0.40\text{mm/h}$ (Fig. S14b). Further experiments find that in the absence of interactions ($K = 0$), the width of the interface between the two colonies is $b_{K=0} = 1.29 \pm 0.02\text{mm}$, and with maximally expressed complementary adhesin pairs the interface width is around $b_{K=max} = 0.50 \pm 0.01\text{mm}$ (Fig. S11).

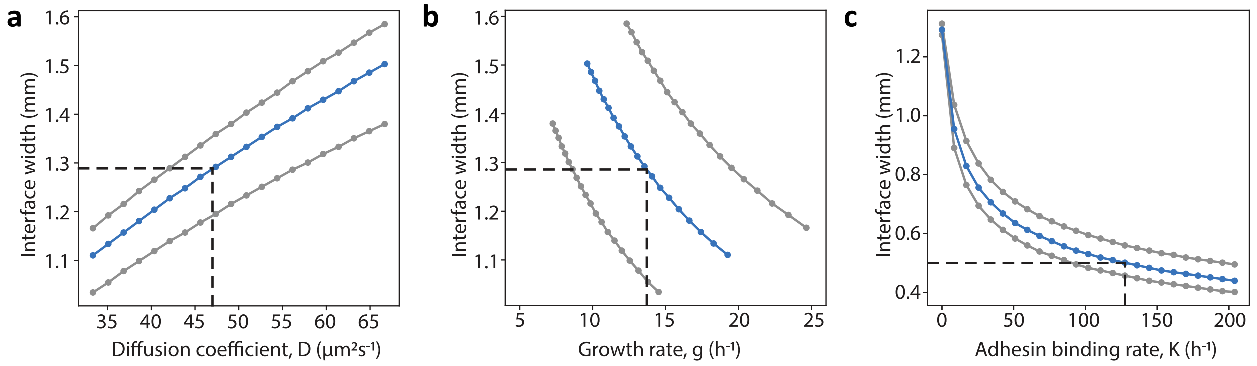

Figure S1: Calibration of model parameters to physical measurements. **(a-b)** To fix the expansion speed, we ensure that $2\sqrt{Dg} = 3.04\text{mm}\,\text{h}^{-1}$, after which we can vary either D or g, (a), (b) respectively (blue dots are simulations). We wish to ensure that the interface width without adhesin ($K = 0$), matches the experimentally measured value of 1.29mm. This constrains the values of $D = 47\mu\text{m}^2\,\text{s}^{-1}$ and $g = 14\text{h}^{-1}$, (black dashed lines). For uncertainty estimates, the same calibration was run with extreme values of the physical parameters to compute upper and lower bounds on D and g (grey dots are simulations with extreme values of physical data as inputs). **(c)** Using the values of D, g, calculated in (a), we vary the adhesin binding rate, K (blue dots are simulations), and use the value which ensures the interface width with adhesin is $b_{K=max} = 0.50\text{mm}$ (black dashed lines). As in (a-b), uncertainty estimates are calculated by running the same calibration with extreme values of the physical data (grey dots).

To calibrate physical parameters, we note that the Fisher-Kolmogorov equations admit fronts that propagate at speed $v = 2\sqrt{Dg}$ [4], which in our model would be the expansion speed of an isolated colony. We therefore ensure that the parameters g, D satisfy $2\sqrt{Dg} = 3.04 \pm 0.40\text{mm/h}$. To calibrate the interface measurements, we performed simulations with initial seeding diameters of $d = 1.5\text{mm}$ and separation $L = 5mm$, running simulations until 65min had elapsed and the interface has formed either with or without interactions, and with identical parameters in both colonies ($g = g_1 = g_2$, $D = D_1 = D_2$). From this, the interface width was measured as the distance between the points where the total amount of species 1, $\rho_1 + \frac{1}{2}\rho_{12}$, is equal to 10% of the bulk value ρ_{max}, and the equivalent point for species 2. A clear length

scale is apparent for the interface width, so other definitions of width will also find a similar value. Simulating without interactions ($K = 0$), for various values of g, D, with $v = 2\sqrt{gD}$ fixed, we find the values $g = 14/\text{h}$, and $D = 47\mu\text{m}^2/s$ ensure that the interface is of width 1.29mm (Fig. S1). To gain uncertainty bounds, we re-calibrated with the extreme ranges of the measured physical parameters, namely with $b_{K=max} = 1.27\text{mm}$, $v = 3.44\text{mm/h}$ and with $b_{K=max} = 1.31\text{mm}$, $v = 2.64\text{mm/h}$, finding that $g = 14 \pm 6/\text{h}$, and $D = 47 \pm 8\mu\text{m}^2/s$ (Fig. S1). Whilst our effective diffusion coefficient agrees with the diffusion coefficient of cells in soft agar ($D = 41.5\mu\text{m}^2/s$ [2]), the effective growth rate is an order of magnitude higher than the experimental measurements (growth rate $a = 0.244 \pm 0.053\text{h}^{-1}$, which we now distinguish from our effective growth rate g). This is due to our minimal model not explicitly modeling chemotaxis-driven expansion. We must therefore have a larger growth rate to achieve the same expansion speed. However, even with these effective parameters, we can accurately predict experimental patterns, Fig. 2.

Next, by including interactions and requiring the interface width to be $w = 0.50 \pm 0.01\text{mm}$, we determine that $K = 130 \pm 50/\text{h}$ (Fig. S1), where again, uncertainty bounds were measured by calibrating with extreme values of the physical measurements. Unless otherwise specified, we use these constants, $D = 47\mu\text{m}^2/s$, $g = 14/\text{h}$, $K = 130/\text{h}$, and $\rho_{max} = 0.155\text{cells}/\mu\text{m}^2$, throughout. Experimentally, some time after an interface forms the chemotactic signal vanishes and cells become non-motile, effectively stopping cell diffusion. Since we do not explicitly model this effect, the model cannot form steady interfaces, and hence we measure the interface width after some finite time elapses, although after formation the subsequent evolution is slow. All simulations were performed using the pseudo-spectral PDE solver Dedalus [5].

In order to simulate two strains which have different expansion speeds, we give each strain its own diffusion coefficient ($D_1 \neq D_2$). By giving each species a different diffusion coefficient D_i, each species will have an expansion speed of $v_i = 2\sqrt{D_i g}$. Therefore, to simulate a strain with half the normal expansion speed, we use a diffusion coefficient $D_i = 0.25 \times 47\mu\text{m}^2/\text{s}$.

The typical interface length scale depends on the diffusion constant D, the effective growth rate g, and the cell adhesion length K. When $K = 0$, dimensional analysis finds that the length scale as $L \sim \sqrt{D/g}$, whereas in the limit of $K \gg g$, dimensional analysis would suggest $L \sim \sqrt{D/K}$. From our fitted values of K and g, these two regimes suggest that the length scale of the adhesin free interface would be a factor of $\sqrt{K/g} \sim 2.7$, larger than the interface with adhesin, indeed experimentally they differ by a factor of around 2.1.

The cell adhesion rate K can also be estimated as follows. The maximum cell density is $\rho_{max} = 0.155 \pm 0.012\text{cells}/\mu\text{m}^2$., so if cells are present at density ρ_{max}, the characteristic separation length scale between cells is $1/\sqrt{\rho_{max}} \approx 3\mu\text{m}$. The typical velocity of cells is around $10\mu\text{m}\,\text{s}$, hence the characteristic time scale for collision will be around 0.3s. Since adhesion occurs after collision, this gives a cell adhesion rate of 3/s, or 10,000/h. This is a factor of 80 higher than the previously fitted value of $K = 130/\text{h}$. This discrepancy occurs as our scaling argument does not account for the fact that

not every collision results in a binding, and that once many cells become bound, it may take significantly longer for any two unbound cells to find each other and collide.

We conclude that the parameters in our model show reasonable consistency with published data as well other theoretical models [2, 6] (see also discussion in supplementary text S1.7).

1.3 Delayed swarm expansion

To simulate unequal seeding ratios, we note that experimentally, cells initially grow with no motility until a critical cell concentration is reached. Diluting the cells experimentally by, say a factor of two, would require the passage of one doubling time to reach the critical cell concentration to initiate swarming. Therefore if one colony starts with N cells, and the other with $2^{-k}N$ cells, the first colony will begin expanding, whilst the second will not until $t = k\tau_{double}$, where τ_{double} is the experimentally measured doubling time, before the cells are motile. In simulations, we therefore allow the first colony to expand starting at $t = 0$, but only allow the second colony to expand starting at $t = k\tau_{double}$, after which the system evolves according to Eq. S1, with results shown in Fig. 2i, with $\tau_{double} = 14.4$min (the doubling rate for Ag2, see section 4.6, which is the strain being diluted).

This captures the experimental behavior of the interface angle as the Ag2 cells are diluted, Fig. 2i, however there is a slight systematic disagreement between theory and experiment. This can be removed by taking into account two additional experimental factors; without dilution Ag2 begins expanding around 10 minutes before Nb2, and that once expanding, Nb2 is slightly faster than Ag2 (0.0506 mm/min vs 0.0491 mm/min, see section 4.6). Recalibrating the effective growth rates using these different speeds, and delaying the expansion of Nb2 by 10 minutes removes the systematic disagreement, Fig S2. However, in the main text and elsewhere in the supplementary, to make a minimal model we use the same parameters for Ag2 as for Nb2, ($g_1 = g_2$, $D_1 = D_2$ in Eq. (S1)), and we calibrate the expansion velocity to that of Nb2. We see from comparing Fig. 2i and Fig. S2, that this will only result in a minor adjustment to the model output.

1.4 Accounting for systematic shifts in interface angle simulations with differential growth and speed

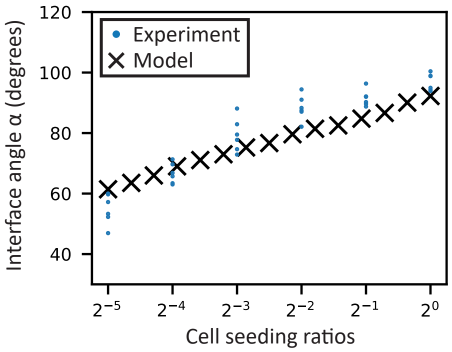

Figure S2: In our minimal model, we simplified the bacterial growth and expansion dynamics by assuming all strains divide and swarm at the same rate. While this is sufficiently accurate in most cases, we noticed a systematic downward shift when tuning the interface angle between swarms in the models (see Fig. 2i). However, experimental measurements suggest a possible difference in growth and expansion rates between strains (Figs. S14, S13). A more accurate model can be achieved by allowing differential growth and expansion rates in the simulation, and applying the experimentally measure values for the relevant strains (Ag2, Nb2) (see Fig S14 for the values). Here, we note that swarming initiates in the 2-3 hour range, and assume a lag phase duration of 1 hour. Applying these adjustments results in a systematic shift upwards and better alignment with experimental measurements. However, for the sake of consistency throughout the paper, in the main text (Fig. 2i) we retain the simplified model.

1.5 Adhesin inhibitor

We model the effect of the adhesin inhibitor as acting on the binding rate through $K = K(I)$, where I is the concentration of the inhibitor. We assume first order Hill-type interaction based on the inhibitor concentration, the half-max inhibitor concentration $I_{1/2}$ and the maximal adhesion $K_{max} = 130/\text{h}$,

$$K(I) = K_{max} \frac{I_{1/2}}{I_{1/2} + I}. \tag{S2}$$

However, to calibrate simulations with the inhibitor to the results in Fig. 2d, we note that whilst the interface width for no peptides added at all is $b_{K=max} = 0.50\text{mm}$, the data with low inhibitor concentration only approaches $b_{K=max} = 0.7\text{mm}$. This discrepancy is due to the fact that for the inhibitor experiment, small peptides were added to the media. It is generally known that changes in media conditions can affect growth rates of bacteria [3, 7], and hence these two values do not match perfectly. Specifically, in these inhibitor experiments a mixture of specific and unspecific inhibitor peptide was added in order to keep total amount of peptide constant and maintain consistency across inhibitor concentrations. Therefore, for Fig. 2d, we first calibrate using $b_{K=max} = 0.7\text{mm}$ and $b_{K=0} = 1.5\text{mm}$, finding that $K = 70/\text{h}$, $D = 67\mu\text{m}^2/\text{s}$, $g = 9/\text{h}$, which we use only for Fig. 2d, and note that these are of consistent magnitude with the previously found parameters. With these new parameters, we find that $I_{1/2} = 5 \times 10^{-6}\text{M}$, when fitting to the experimental data in Fig. 2d.

1.6 Multi-species extension

Our framework readily extends to multiple adhesion pairs. For instance, if species 1 and 2 formed an adhesion pair as did 3 and 4, then the density fields would evolve as

$$
\begin{aligned}
\frac{\partial \rho_1}{\partial t} &= g_1\rho_1 c(\bar{\rho}) + D_1\nabla^2\rho_1 - \tilde{K}_1\rho_1\rho_2, \\
\frac{\partial \rho_2}{\partial t} &= g_2\rho_2 c(\bar{\rho}) + D_2\nabla^2\rho_2 - \tilde{K}_1\rho_1\rho_2, \\
\frac{\partial \rho_3}{\partial t} &= g_3\rho_3 c(\bar{\rho}) + D_3\nabla^2\rho_3 - \tilde{K}_2\rho_3\rho_4, \\
\frac{\partial \rho_4}{\partial t} &= g_4\rho_4 c(\bar{\rho}) + D_4\nabla^2\rho_4 - \tilde{K}_2\rho_3\rho_4, \\
\frac{\partial \rho_{12}}{\partial t} &= 2\tilde{K}_1\rho_1\rho_2, \\
\frac{\partial \rho_{34}}{\partial t} &= 2\tilde{K}_2\rho_3\rho_4, \\
c(\bar{\rho}) &= 1 - \bar{\rho}/\rho_{max} = 1 - (\rho_1 + \rho_2 + \rho_{12} + \rho_3 + \rho_4 + \rho_{34})/\rho_{max},
\end{aligned}
\tag{S3}
$$

where now the strength of adhesion, $\tilde{K}_1, \tilde{K}_2$ could vary across different adhesin pairs. To represent a mixture of, for instance, species 1 and 3, we seed both 1 and 3 in the same location, each at density $\rho_1 = \rho_3 = 0.5\rho_{max}$, so that locally $\bar{\rho} = \rho_{max}$, and they will remain mixed as the simulation evolves. An example with 3 species, where species 1 and 2 form an adhesin pair, and species 3 does not interact with either, and initial seeding conditions mix species 2 and 3, as experimentally realized in Fig. 3, is shown in Fig. S3. This can be similarly extended to 3 adhesin pairs, as was done in section 4.20.

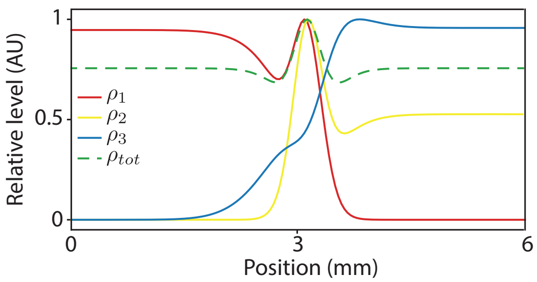

Figure S3: Simulated interface between three species, reproducing the experimental results in Fig. 3b. Species 1 (red) and 2 (yellow) form an adhesin pair, whilst species 3 (blue) does not interact with either of them. Species 1 was initially seeded to the left, whilst a mixture of species 2 and 3 was seeded to the right, and the separation between seeding sites was 5mm. The simulation was run until 65min had elapsed.

1.7 Comparison to other models

Mathematical models describing the expansion of bacterial populations have been widely used [8–10], including the Fisher-Kolmogorov equation [2, 4]. Whilst Fisher-Kolmogorov dynamics are sufficient to describe fronts propagating at a fixed velocity, additional terms are required to model branching instabilities [8, 9], or exponential growth [10]. Cremer et al. [2] proposed a model with additional nutrient and attractant fields, which match experiments both in expansion dynamics and density profile. They also compared against Fisher-Kolmogorov dynamics, finding that the expansion dynamics broadly agree for times less than 5h [2], which describes the relevant regime for the experiments analyzed here. Whilst the Fisher-Kolmogorov equation leads to a less realistic profile of a freely expanding front than a model with nutrient and attractant fields [2], we find that, together with the adhesion terms, our model does reproduce well the stationary interface between two colonies, Fig. 2. Therefore, to find a minimal model which reproduces the expansion and interaction behavior seen in experiments, we use a simple Fisher-Kolmogorov type dynamics with adhesion term, and do not explicitly model the nutrient or attractant fields as it is beyond the scope of the current work.

In the next subsection we provide an example for such a model extension.

1.8 In-silico extension of model to recapitulate multiple chemotactic waves

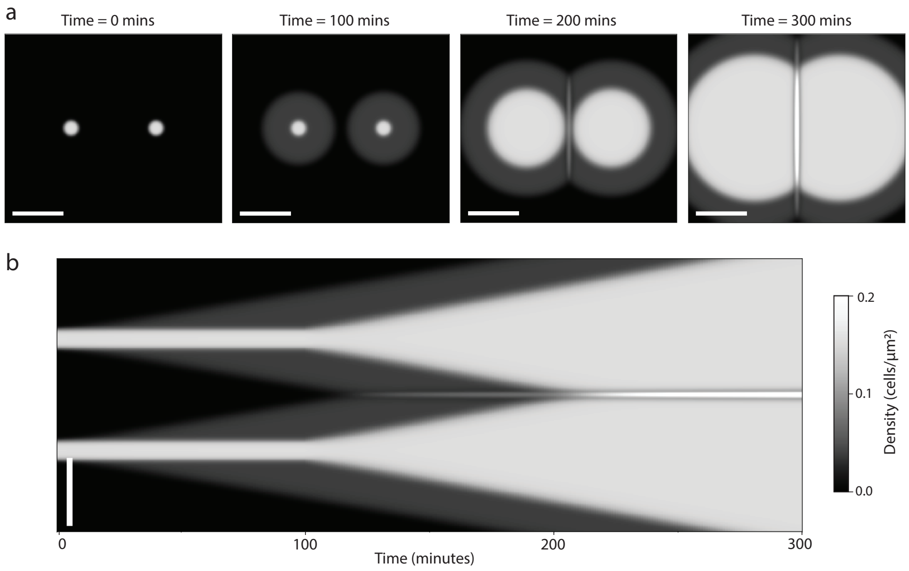

Figure S4: Simulation of model supporting multiple chemotactic waves. **(a)** Snapshots of simulation with two colonies seeded 9mm apart. We take time $t = 0$ min, to be the point when the first chemotactic wave starts expanding, with the second chemotactic wave starting at $t = 100$ min. By $t = 200$ min, the first wave has already started to form an interface, and by $t = 300$ min, both waves have met in the middle and formed the final interface. Scale bar is 5mm. **(b)** Kymograph of the simulation using the horizonal line drawn through the centers of the two initial colonies. We can see that the first expansion wave begins at $t = 0$ min, whereas the second starts at $t = 100$ min. Both have similar slopes indicating expansion occurs at the same speed for each wave. An initial interface begins to form at around $t = 130$ min, but the full interface does not form until around $t = 230$ min, when the second chemotactic wave reaches the interface. Scale bar is 5mm.

While the model in Eq. (S1) represents a minimal model that quantitatively reproduces many aspects of the experiments, it does not capture all behavior. In particular, the growth of colonies is experimentally observed to occur through multiple chemotactic waves (Video S1), whereas the minimal model assumes that this growth occurred through a single expansion wave. These waves occur as a result of cells specializing to consume a specific nutrient, with these specialized cells differing in their growth rate, resulting in multiple waves as some cells start expanding before others [11, 12]. Other theoretical models of expansion also have focused on the case of a single expansion wave [2, 10], however it is straightforward to extend our model to the case of multiple waves.

To include this behavior into our model, we consider the case of two different chemoattractants, u and v, giving rise to different cell subpopulations, ρ_i^u and ρ_i^v, which begin swarming at different times. Once expansion starts, the velocity

does not differ significantly between waves, so we assume the same effective growth rates g, and diffusion constant D for both subpopulations. As these subpopulations consume different nutrients, the maximum density of each subpopulation differs and is given by ρ^u_{max} and ρ^v_{max}, which combine to give the total measured maximum density, ρ_{max}. In total, the model in Eq. (S1) becomes,

$$
\begin{aligned}
\frac{\partial \rho_1^u}{\partial t} &= g\rho_1^u c^u(\bar{\rho}^u) + D\nabla^2\rho_1^u - \tilde{K}\rho_1^u\rho_2, \\
\frac{\partial \rho_1^v}{\partial t} &= \begin{cases} g\rho_1^v c^v(\bar{\rho}^v) + D\nabla^2\rho_1^v - \tilde{K}\rho_1^v\rho_2 & t > t_c \\ 0 & t < t_c \end{cases}, \\
\frac{\partial \rho_2^u}{\partial t} &= g\rho_2^u c^u(\bar{\rho}^u) + D\nabla^2\rho_2^u - \tilde{K}\rho_1\rho_2^u, \\
\frac{\partial \rho_2^v}{\partial t} &= \begin{cases} g\rho_2^v c^v(\bar{\rho}^v) + D\nabla^2\rho_2^v - \tilde{K}\rho_1\rho_2^v & t > t_c \\ 0 & t < t_c \end{cases}, \\
\frac{\partial \rho_{12}}{\partial t} &= 2\tilde{K}\rho_1\rho_2, \\
\rho_1 &= \rho_1^u + \rho_1^v, \\
\rho_2 &= \rho_2^u + \rho_2^v, \\
c^u(\bar{\rho}^u) &= 1 - \bar{\rho}^u/\rho^u_{max} = 1 - (\rho_1^u + \rho_2^u + \rho_{12})/\rho^u_{max}, \\
c^v(\bar{\rho}^v) &= 1 - \bar{\rho}^v/\rho^v_{max} = 1 - (\rho_1^v + \rho_2^v + \rho_{12})/\rho^v_{max}, \\
\rho_{max} &= \rho^u_{max} + \rho^v_{max}.
\end{aligned}
\tag{S4}
$$

We simulate this model with $\rho^u_{max} = 0.25 \times \rho_{max}$, $\rho^v_{max} = 0.75 \times \rho_{max}$ and $t_c = 40$min, to approximately match to experimental observations. We set D, $\tilde{K}$, and g to be the same as the previously calibrated parameters, which were calibrated to the second and largest expansion wave. As the expansion speeds are comparable, the initial wave and the main wave will end up forming an interface in the same place (Fig. S4, Video S6). As the final interface pattern is unaffected by the presence of the first wave, elsewhere we use the model of Eq. (S1), which provides a minimal model of the dynamics.

1.9 Analytical model of interface angles with differential growth and expansion

To gain intuition into the determinants of interface geometry, we developed a simple analytical model of our experimental platform. The shape of the interfaces between two expanding colonies can be modeled by starting at each seed, drawing successively larger circles, and defining the interfaces at the points the circles intersect.

To arrive at an analytical model, we begin with the general form for the equation of a circle:

$$(x - x_0)^2 + (y - y_0)^2 = r^2$$

Now, consider two strains seeded in the Cartesian plane, with strain 1 seeded at the origin, and strain 2 seeded at $(x_p, 0)$, where x_p is the seeding distance between the two strains. Now, the following pair of equations describes the circles drawn by the two strains:

$$x_1^2 + y_1^2 = r_1^2$$
$$(x_2 - x_p)^2 + y_2^2 = r_2^2$$

Where the subscripts indicate strains 1 and 2.

At any point in time, we know the radii of the circles given the time elapsed (t) and the expansion speed (k). We can also account for the delay in initiation of swarming by introducing a delay term τ. Altogether:

$$r_1 = k_1(t - \tau_1)$$
$$r_2 = k_2(t - \tau_2)$$

However, we can further simplify this by assuming strain 2 has a longer delay than swarm 1, defining $\tau = \tau_2 - \tau_1$, and restricting the model to $t > \tau_1$. For simplicity, we restrict the model to $t \geq \tau_1$, and with some abuse of notation we arrive at the following:

$$x_1^2 + y_1^2 = r_1^2 t^2$$
$$(x_2 - x_p)^2 + y_2^2 = k_2^2(t - \tau)^2$$

Finally, we note that the interface is defined by the points where $x_1 = x_2 = x$ and $y_1 = y_2 = y$. After substituting and solving for x and y, we find the interface shape is described analytically with the following set of parametric equations (Eq. S5):

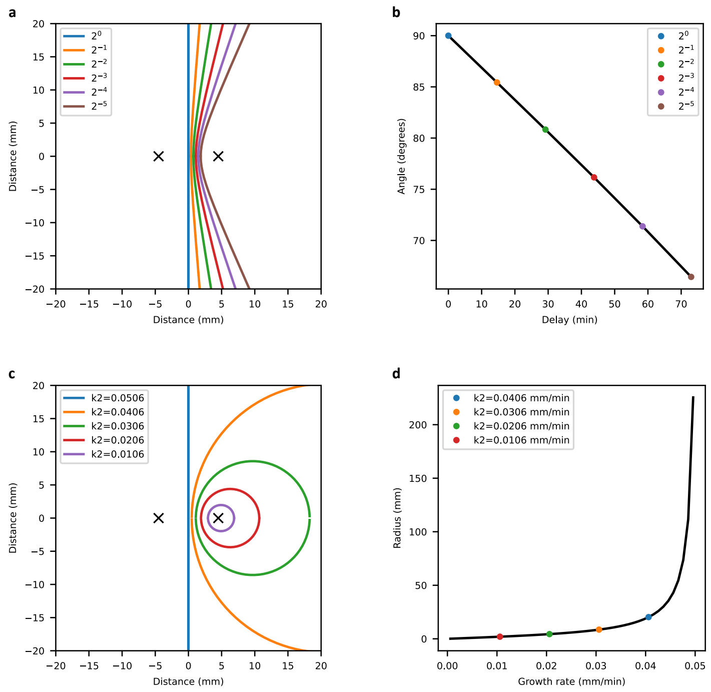

Figure S5: Analytical model of variable growth geometries. Per experimental measurements, $k_1 = k_2 = 0.0506$ mm/min and $x_p = 9$ mm. **(a)** Interface profiles with increasing delay τ, while keeping k_1, k_2, and x_p constant. **(b)** Fits to interfaces as in (A) shows a roughly linear relationship between delay τ and interface angle. See also Fig. 2i. **(c)** Interface profiles with decreasing expansion rate k_2, while keeping k_1, τ and x_p constant. **(d)** Radius of interfaces as a function of differential expansion rate.

$$\begin{aligned} x &= (x_p^2 + k_1^2 t^2 - k_2^2 (t-\tau)^2)/2x_p \\ y &= \pm\sqrt{k_1^2 t^2 - x^2} \end{aligned} \tag{S5}$$

Where k_1 and k_2 are the expansion speeds of the strains, τ is the delay before initiating expansion for the two strains, and x_p is the initial seeding distance between the strains which are seeded along the $y = 0$ axis. Measurements of the expansion rates between our Ag2, Nb2, Ag3, Nb3 and null lines show all strains expand at similar rate of 0.0506 ± 0.0067 mm/min, with a doubling time of 14.6 ± 3.2 min (see supplementary text S4.6). x_p was experimentally fixed at 9 mm.

The analytical solution for cells growing at the same rate $k_1 = k_2$ is a straight line perpendicular to the axis defined by the two seeding points (Fig. S5A). However, introducing a delay $\tau > 0$ into the initial timepoint of cell expansion causes this line to deviate from perpendicular at some angle (Fig. S5a) that linearly scales with the delay time (Fig. S5b). See also the experimentally and phase-field model derived relationship shown in Fig. 2f,i.

Varying the expansion rates ($k_1 \neq k_2$) results in the faster growing colony engulfing the slower colony (Fig. S5c) in a

circular interface geometry. The greater the expansion rate difference, the smaller the radius of the engulfed region (Fig. S5d). See also experimental and phase-field models in Fig. 2g.

2 Text S2: Mathematical and Algorithmic Aspects of Interface Pattern Formation

In this section we first provide some general background on related tiling, tessellation and map coloring problems. We then classify some mathematical problems that are motivated by our observations and results, and which ultimately allow us to systematically prove that four adhesins are sufficient from a mathematical point of view to generate any arbitrary interface pattern. Finally, we discuss solutions to more specific patterning cases with certain constraints. This section also motivates open questions for future mathematical research.

2.1 Tiling, tessellation and map-coloring problems; definitions

Geometric tiling, tessellation, map coloring, and related problems have a long history; see for example [13–18]. Here we briefly review a few concepts of special interest for the present work.

The primary literature (as well as other informal resources) often use the terms 'tiling' and 'tessellation' interchangeably, and when definitions are provided they are not necessarily consistent. We find the following concepts appropriate:

We define **map** in the following context as a space filling division of the plane.

A **tiling** starts with a countable set of geometric elements and one then aims to completely fill a space with those elements without gaps or overlaps. A formal definition is provided by [17]: "A plane tiling is a countable family of closed sets $T = \{T_1, T_2, ...\}$ which cover the plane without gaps or overlaps." Hence the classic tiling problems raise the question whether and how specific tile geometries (one or multiple tile types) can completely fill a space, and whether specific complete or partial symmetries emerge in this larger pattern [17] (Fig. S6a). Reference [17] provides a detailed overview of the field, and [18] provides a more recent perspective and update.

A **tessellation** starts with a number of seeding positions distributed in space from which (according to certain rules) interfaces between seeding positions are constructed that ultimately lead to a space-filling division of this space into compartments. An example of a formal definition of the Voronoi tessellation is provided by [19]: "The Voronoi diagram (also commonly referred to as Dirichlet tesselation, or Thiessen polygon) of a set S of n points is a PSLG [planar straight-

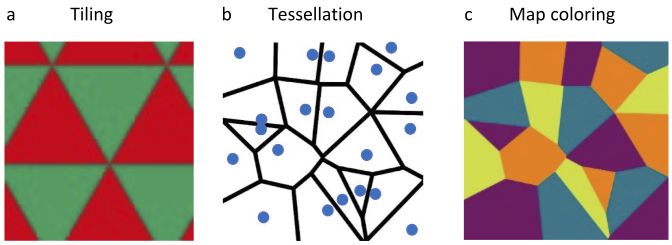

Figure S6: Examples tiling, tessellation, and map coloring.

line graph] with n cells, where each cell belongs to one point from S and consists of all points in the plane that are closer to that point than to any other in S." Generalizations exist, e.g., the lines may be defined by some other metric instead of the closest point, and those lines also do not have to be straight - using bacteria with different growth rates as demonstrated in the main paper in Fig. 2g provides an illustrative example. Here the term 'line' is synonymous with the term 'interface' in the main paper. The inverse Voronoi problem seeks to determine the position of these points S given a PSLG - but this is not possible for every PSLG. However, it can be shown that the interfaces of any given pattern are the subset of some other Voronoi tessellation pattern [20]. This implies that if enough originating points are used and if some interfaces can be hidden afterwards, any pattern of interfaces can be generated that way. Finding the positions of the set S in this case is also termed the 'Generalized Inverse Voronoi Problem (GIVP)'[21–23].

Note that the concepts of tiling and tessellation have significant overlap. For example, a tessellation problem on a regular grid (or at least where the grid points have certain generative properties) will lead to a corresponding tiling problem, but not every tessellation corresponds to a tiling. Similarly, a tiling problem where every tile can be generated through a tessellation process corresponds to tessellation problem, but not every tiling corresponds to a tessellation.

The **four-color map theorem** states that only four different colors (or attributes) are required to ensure that no adjacent tiles of any map in the 2D plane have the same color [14, 15, 24] (Fig. S6c).

2.2 Classes of interface patterning problems and their general solutions

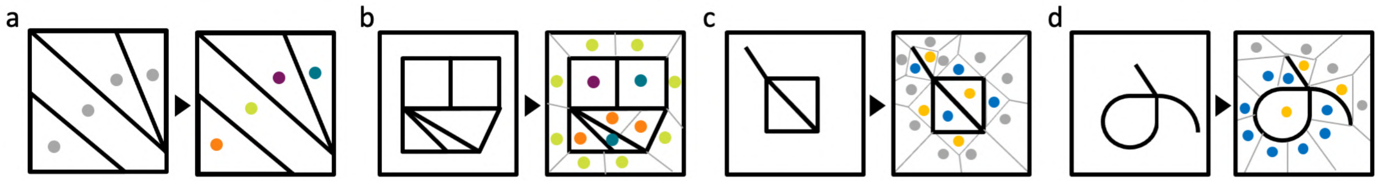

Figure S7: Illustration of interface patterning problem classifications with increasing complexity. **(a-d)** Problems 1-4 (see text).

In the following we now introduce a classification of possible interface patterns with increasing complexity. This classification is motivated by the different algorithms discussed in the paper that systematically generate patterns of increasing complexity. The logic and mathematical problems presented here could of course be implemented by various physical means other than adhesive bacteria, and they therefore would apply much more general to other systems. For simplicity, we use language as if all interface patterns are generated through bacteria that swarm out from various point sources ('organizers') across a plane. Wherever now any two such bacterial strains meet they may or may not form a distinct and visible interface based on pairwise adhesive properties of these strains. Furthermore, they stop each other from moving beyond this interface. This motivates several questions. What type of interface patterns can be generated in general? What types of and how many adhesins are required? At what positions do they need to be placed in order to generate a desired pattern? We do not consider any experimental limitations due the non-finite size of seeding position, or the precision and accuracy associated with any liquid handling device; and hence we assume that the diameter of any seeding position and the width of any interface are sufficiently small compared to feature sizes of the desired pattern. Overall, this also represents a new class of problems that is distinct from the classic tiling and tessellation problems (see above), as it adds the question of whether any particular interface is generated or not (i.e., 'visible' vs. 'hidden'), where interfaces also can have open ends (Fig. 4d). Hence this problem is not only about generating a map of tiles, but also about generating a pattern (or 'graph' or 'network') of interfaces.

We can classify the desired patterning problems in four levels of increasing complexity (Fig. S7), which are primarily chosen as the key steps to prove that four adhesins (i.e., two adhesin pairs) are sufficient to generate any arbitrary interface pattern. Other classifications and more specific scenarios are also possible.

1. Voronoi tessellation; interface pattern without open interfaces

Problem: Given is a desired 2D tiling in the plane with 'interfaces' (boundaries) being present between any two adjacent tiles, where this tiling corresponds to a Voronoi tessellation, i.e., the seeding positions ('organizers') are predetermined at the proper location, and all bacterial strains have identical growth and movement properties (Fig. S7a). What adhesin

combinations are required at each seeding position, and how many adhesins are required in total, in order to generate this pattern?

Solution: The solution is detailed and solved in the main paper (Fig. 4b) and corresponding text. The solution essentially rests on utilizing the four-color map theorem.

2. Arbitrary interface positioning; but no interface with open interfaces

Problem: As before, but now the seeding positions have not yet been identified, and the desired pattern is not necessarily a Voronoi tessellation, but both ends of each interface still end at another interface (Fig. S7b). Where should organizers be placed, what adhesin combinations are required at each organizer, and how many adhesins are required in total, in order to generate this pattern?

Solution: A complete mathematical solution is provided in the main paper (Fig. 4c) and corresponding text. The solution essentially rests on solving the Generalized Inverse Voronoi Problem (GIVP), utilizing the four-color map theorem, and making all newly introduced interfaces hidden by always using the same adhesins combinations for all seeding positions within any original tile.

3. Arbitrary interface positioning; interfaces with open ends are possible

Problem: As in previous problems, but now we also allow for 'open interface patterns', i.e., the ends of an interface do not have to end at another interface, hence interface patterns can emerge that are not found in classic tiling or tessellation problems [17] (Fig. S7c). This includes the set of all straight-line interface patterns. Question as in previous problem.

Solution: One needs to introduce hidden interfaces (see Fig. 4c, d). Different algorithms are possible, for example:

(1) An algorithm as detailed in the paper: Using the four doublet strains (Fig. 3d) together with a null strain while minimizing hidden interfaces, and where then also the Generalized Inverse Voronoi Problem needs to be solved.

(2) A (semi-trivial) algorithm can be achieved with a single adhesin pair and just three strains (two with one adhesins each and a third with the null element), and where on each side of each interface one of two complementary adhesins are placed. Then a large number of 'hidden triangles' is added at any place where an undesired interface would form. This algorithm has the potential downside of requiring in general a larger number of seeding positions. (Note that a completely trivial extension of this algorithm is to just 'draw' lines with either adhesin on both sides of any desired interface, and which is then further sandwiched by two lines with the null strain. In this case there is no emergent patterning process happening, instead the desired interface pattern is essentially already drawn 1:1.)

4. Most general case

Problem: As in previous problem, but now we also allow for all sorts of extensions. For example, we now allow curved interfaces, or other more complex features (Fig. S7d). Question as in previous problem, but now extended to various other dynamic and biophysical properties that can be associated with these adhesins and thereby affect the positioning and geometry of these interfaces. Further extensions are represented by environmentally dependent logic, as illustrated in figure 4g.

Solution: The paper provides an illustration for one specific pattern in figure 4f. The accompanying continuum model (Fig. 2b) allows for quantitative exploration of pattern spaces prior to experimental implementation.

Here an iterative algorithm could be implemented that guesses a first seeding position, models the outcome, determines how close the match is (based on a user-chosen metric), and then updates the seed. The solution is not unique, and with sufficiently many seeding positions an arbitrary shape can be approximated arbitrarily well. Constraints would also have to be decided upfront, e.g., whether the number of seeding positions should be minimized, or within what resolution and error the achieved pattern should match with the desired one.

A deeper analysis and classification is beyond the present work. But in the following subsection we also provide solutions to some more specific cases that all fall under problem 3 (or even problem 2).

2.3 Solutions to more specific interface patterning problems

Regular periodic tilings with seeding positions at circumcenters, all interfaces visible

For square and triangular tillings one adhesin pair is sufficient, for hexagonal tilings two adhesin pairs are required as illustrated in figures 4a and Fig. S26. A hexagonal tiling cannot be achieved with one adhesin pair as three tiles border each other in a pairwise manner, and as neighboring tiles need to have different adhesins in order to form an interface. In general, multiple seeding configurations can lead to the same interface pattern, as illustrated with a hexagonal tiling which can be achieved with both three or four of the adhesin doublets (Fig. S8a).

1-D chain of square tiles with seeding positions predefined at circumcenters, hidden interfaces possible

If tiles form a 1D chain (either being infinite or bounded on each side), a single adhesin pair is sufficient to generate any desired interface pattern by linearly going through the tiles - always adding the complementary or the same adhesin to the next tile, depending on whether an interface is or is not desired between adjacent tiles, respectively (Fig. S8b). Note that for a 1-D chain with periodic boundary conditions (i.e., the last tile on one end directly connecting to the first tile on the other end forming a closed loop) one adhesin pair is generally not sufficient, as the last interface to be generated cannot be chosen freely, but instead is automatically determined (Fig. S8c, red arrow indicating the inconsistency). Even including the null element as a third strain cannot always resolve this issue, although in some scenarios it can.

Minimally required number of adhesin pairs for regular 2D tilings, seeding positions predefined at circumcenters, hidden interfaces possible

In general, at least two adhesin pairs are required to generate every possible interface pattern for any 2D tiling, as one can easily construct a 'closed loop' with one inconsistent interface (similar to the closed 1-D chains shown in figure S8c with the red arrows marking the inconsistencies). Should no closed loop exist at all - then this in not truly a 2D tiling, instead it is a quasi 1D tiling without periodic boundary conditions.

In the case of **triangular lattices**, we found an algorithm that requires only two adhesin pairs (Fig. S8d, Fig. 4e main paper): (i) Go row by row. For all odd rows use adhesin pair 1, for even rows use pair 2. (ii) In each row, pick a starting triangle and add one random adhesin, and then go from left to right (and then also right to left to fill other half plane): if an interface is desired between horizontally adjacent triangles then switch to the complementary adhesin partner, otherwise keep the same adhesin. (iii) Finally fill in all desired horizontal interfaces by considering only those triangles that point their tip downwards. If an interface above that triangle is desired, then add the complementary adhesin to this triangle in regards to the adhesin already being in the triangle above. As the last step does not influence interface formation within any row, every desired pattern can be achieved with this algorithm. Note that this algorithm uses up to eight different strain mixtures (the four single adhesin strains, and the four pairwise adhesin strains). Note also

that finding a solution to a desired pattern scales linearly with the number of tiles. (The **pattern shown in Fig. 4e** is surrounded by the null elements for convenience, but the null element is not necessary, i.e., based on the above algorithm the same could be achieved with the adhesive strains alone. The **pattern shown in Fig. 1d-f** could have also been established with the algorithm described here, but instead was determined by trial and error while making use of multiple symmetries not present in the general case; therefore it has a different seeding structure and which again emphasizes that

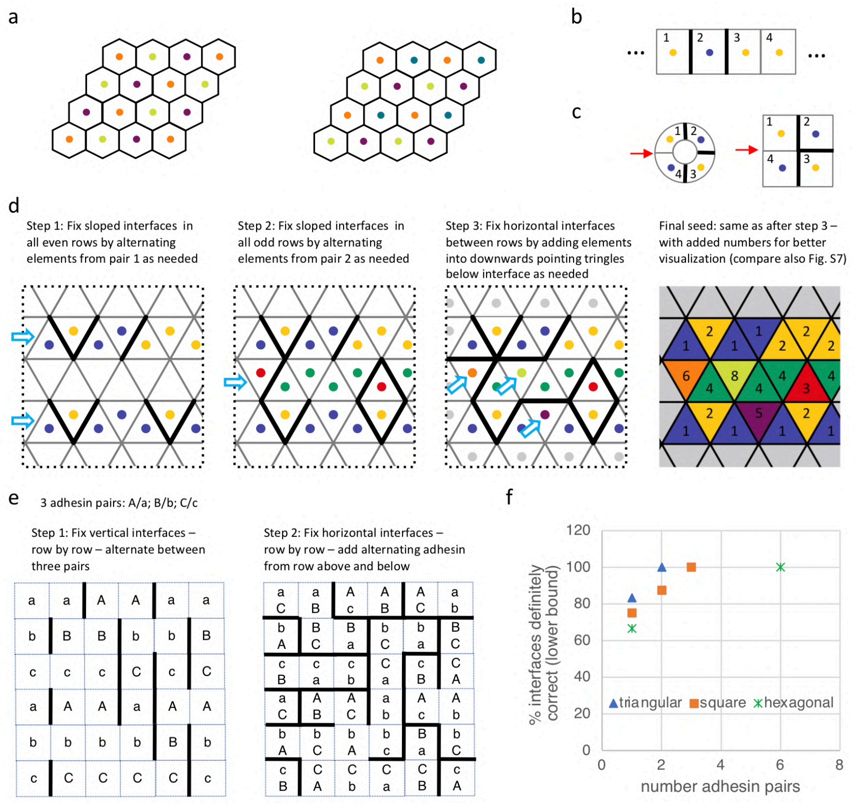

Figure S8: **(a)** Demonstration that different seeds can lead to the same interface patterns as illustrated by realizing a hexagonal lattice with both three or four adhesin doublets. **(b-e)** Algorithms for generating interface patterns with seeding positions predefined at circumcenters and hidden interfaces permitted: (b) 1-D open chain can be generated with single pair by alternating between adhesins as needed; (c) 1-D chains forming closed loops cannot be achieved in general with a single pair - red arrow pointing to the frustration, frustration can be easily resolved with second pair; (d) 2-D triangular lattice can be solved with two pairs; (e) 2-D square lattice can be solved with three pairs (letters instead of colors denote the three pairs, i.e., A/a B/b C/c). **(f)** Given number of available adhesin pairs and any desired arbitrary pattern on a regular lattice with both hidden and visible interfaces, what percentage of interfaces can be forced to have the desired property of being visible or hidden? The values represent lower bounds; future algorithms might perform significantly better. See text for details. Color coding as in main paper.

the seeding patterns are not unique.)

In the case of **square lattice** we found an algorithm with a similar idea that requires only three adhesin pairs (as illustrated in Fig. S8e). We consider rows modulo 3, each of which is patterned horizontally (i.e., selecting for the vertical interfaces) using pair 1, 2, and 3 respectively. The horizontal interfaces are then filled in by going along each row and alternating between adding the complementary adhesin to what is in the row above or in the row below - in case any particular interface should be formed. We did not identify an algorithm that requires only two adhesin pairs, and we conjecture that none exists.

In the case of the **hexagonal lattice** we found an algorithm using six adhesin pairs: Each of the three main directions is solved with two different adhesin pairs with the 1-D chain approach (Fig. S8c). Along any direction, the odd rows are solved with the first of the two adhesins, the even rows with the second adhesins. This leads to using three different adhesins per hexagon, and three adhesin pairs in total. Potentially other algorithms exist that require fewer adhesins.

Note that for an infinite lattice, one can always start at an arbitrary tile, and then pursue these algorithms into all directions independently.

In **conclusion**, for the regular triangular tiling this proves that two adhesins are necessary as well as sufficient to generate any arbitrary interface patterns, i.e., where every interface can be selected to be visible or hidden. For the square and hexagonal tiling we now identified a lower vs. an upper bound, i.e., two vs. three and two vs. six adhesin pairs, respectively. We have not yet identified algorithms in these cases that would have a lower upper bound on required adhesin numbers; the reader is invited to search for those. Note that it is the restriction of seeding point positions that ultimately prevents using the GIVP and four-color map approach to directly restrict the number of required adhesins to four.

Regular 4xN rectangular or triangular lattice, seeding positions predefined at circumcenters, hidden interfaces possible

For a 4xN square lattice, it is possible to form any pattern using only two adhesin pairs. This lattice is interesting as it corresponds to the commonly used 7-segment digital display, and as adding additional triangles in the two middle rows a 16-segment display can be generated (Fig. 4h). These problems can then be solved with just two adhesin pairs, as they are dealing essentially with two 1D chains of tiles, where each chain can be solved with one adhesin pair, and where the lines between the rows can be forced by adding additional adhesins of the opposite type in rows above and below. Note that this algorithm may require all nine possible strain combinations (Fig. 3d). For more rows this algorithm does not work in general.

Patterning complexity vs. number of available adhesins; regular tilings and hidden interfaces possible

As we saw, if the number of adhesin pairs is limited, not all possible interface patterns can be generated. But this raises the question: "Given a set of seeding positions and a specific number of adhesin pairs, what fraction of all interfaces can certainly be instructed to be either visible or hidden (while the remaining interfaces cannot be forced)?" (Note that this provides one way of quantifying the overall patterning complexity, but there are certainly other ways of doing so.)

For example, in the case of the **1-D chain** consisting of N tiles and which has periodic boundary conditions (Fig. S8c, d), only one of these interfaces cannot be determined at will, which implies that as N goes to infinity, the fraction of properly determined interfaces approaches 1.

In the case of the **triangular lattice** that fraction is at least 5/6 for one adhesion pair, which can be seen as follows: Moving along any given horizontal row all interfaces in that row can be determined as described above, which is 2/3 of all interfaces. The row below is then determined again the same way, again fixing 2/3 of these interfaces correctly. But at the same time, the remaining 1/3 of the interfaces between these two rows are now determined at random. In case at least half of these interfaces are determined as desired, one continues with the next row the same way. In case less than half of interfaces between both rows are correct, then one just swaps in the lower row the two adhesin from that pair, which will have no affect for any interface within that row while all interfaces between rows will now swap, i.e., hidden interfaces become visible and visible ones become hidden. This has the effect that whatever the fraction of correctly determined interfaces between rows was before, it is now 1 minus that fraction. Hence at least half of all interfaces between any two rows can be forced as desired. In conclusion, that leads to $5/6 = 2/3 + 1/2 * 1/3$ of all interfaces can be determined.

Similar strategies lead to 3/4 and 2/3 of all interfaces for one adhesion pair for the **square and hexagonal lattice**, respectively. The reader is invited to check.

We do not know whether algorithms exist that can do better than this for one adhesin pair, and we do not know what the corresponding limits for square and hexagonal lattice are if more than one adhesin pair can be used (see also Fig. S8f for a summary for best performing algorithms identified so far). We conjecture that for square lattice and two adhesin pairs an algorithm exist that allows one to fix at least 7/8 of all interfaces. The reader is invited to identify algorithms that perform better.

How many bits are truly utilized in the demonstrated adhesion logic?

As illustrated in Fig. 3d and associated text, utilizing any of the four different adhesins provides in principle 16 possible combinations, which corresponds to $log_2(16) = 4$ bit of information, i.e., '4-bit adhesion logic'. In practice, not all combinations are possible due to self-interaction among the complementary adhesion pairs. Specifically, out of the $2^4 = 16$ combinations, 7 have to be excluded (the quadruplet, all triplets, and the two doublets consisting of complementary adhesins) due to self-interaction, which leaves 9 possible combinations corresponding to $log_2(9) = 3.16$ bits. We also

showed that general interface patterns with open ends can already be achieved with just the four doublets and the null, leading to $log_2(5) = 2.32$ bits. Patterns without open ends can be achieved with just the four doublets, leading to $log_2(4) = 2$ bits. Future natural or synthetic adhesin systems may potentially utilize these self-interactions in interesting ways for spatial patterning and would thereby utilize all four bits.

Adhesion logic with more than 4 adhesins

Using more than 4 adhesins (4-bit strategy) is possible in principle (see simulations in Fig. S28), which leads to tangible benefits and also potential limitations. Benefits include the need for fewer seeding positions, and it was also explicitly shown that certain patterns on a square lattice with prefixed seeding positions are achievable with six adhesins but not with four (Fig. S8e, f).

Practical (experimental) limitations include the challenge that with increasing number of strains on a given seeding position the strength of the individual adhesive interactions is diluted and the formed interfaces are potentially weakened, e.g., when seeding 3 different adhesins at the same position instead of just 2. But note that such limitations could be avoided, for example, when using a 6 bit adhesin code for the overall pattern while still never using more than two adhesins per seeding positions (Fig. S8e).

3 Text S3: Practical Considerations for Applying Adhesin Logic as a Biosensor

The application of living cells as whole-cell biosensors is attractive due to the potential for low-cost, portability and robustness [25, 26]. However, there are still very few examples of commercially implemented whole-cell biosensors [26], highlighting the relative novelty of this approach and the corresponding challenges. Here we discuss some considerations for applying soft agar based adhesin logic as a whole-cell biosensor.

Time to result: The time it takes for the interface readout can be estimated with the following equation:

$$T = \tau_0 + dv$$

Where T is the time to result, τ_0 is the delay for the onset of swarming, d is the seeding distance, and v is the average speed of the two swarms.

The distance d can easily be tuned by designing the seeding positions to be closer or further apart. However, we have found that with a standard pipette and 0.5-1μl volumes, 1.5mm is the minimum practical seeding distance due to the finite size of the droplet and non-motile expansion of the initial seeds at early time-points. τ_0 can be tuned by adjusting the seeding density, as well as the storage conditions of the inoculate. Both τ_0 and v can be tuned by adjusting the temperature of the assay. Experimentally, we found that τ_0 requires approximately 2 hours for the bacteria to go through lag phase and a few cell divisions when inoculated from saturated overnight cultures.

Stability and storage: Standard 1.5% agar plates with antibiotics can be stored sealed at 4°C for up to a month. We expect similar performance for soft agar. However, soft agar is succeptible to moisture loss, and is significantly more mechanically fragile than standard 1.5% agar plates. Rough handling, and prolonged storage at an angle could disrupt the mechanical integrity of the gel. However soft agar gels are very easy to make from powder, which has a long shelf life at room temperature. While autoclaving is the preferred method, we have found that microwaving the suspended powder in water produces a gel that is comparable to the autoclaved product.

Susceptibility to variance in environmental conditions: The assay is relatively robust to environmental conditions. The most important environmental variables for the soft agar assay are humidity and temperature. For humidity, it is sufficient to keep the assay locally humidified. For example, standard petri dish with the lid on inside of a unhumidified incubator is sufficient. However, it is also important that the cells have access to oxygen, so the assay cannot be airtight.

For temperature, we have found that 37°C works well, although we noticed certain strains form stronger interfaces at room temperature. On the other end of the spectrum, temperatures exceeding 37°C results in unpredictable self-clumping behavior even in the absence of adhesins. Regardless, the assay is ideally designed for and run at some optimal

temperature, as relative growth and migration dynamics between strains can be differentially impacted by temperature fluctuations.

Susceptibility to colony-to-colony variation: With consistent protocols in storing and inoculating cultures, colony-to-colony variations do not have a significant impact on the assays. However, consistent protocols may not be possible in an application setting. In general, while the formation of visible interfaces is robust to changes in conditions, the location and curvature of the interface can be strongly affected. For this reason, complex patterns may be undesirable in applications where culturing conditions cannot be well controlled.

Susceptibility to variance in metabolic load: The bacterial strains reported here had roughly comparable growth and migration rates. However, this may not always be the case, especially when designing more complex circuits with heavier metabolic loads. In these cases, computational tools could potentially be used to predict and aid design of gene circuits [27]. If these approaches fail, computational simulations like those presented here could be utilized to rapidly iterate over seeding designs in order to accommodate differences between strains.

Persistence of pattern: The interfaces shown in the paper are stable long-term. Stored at 4°C, the patterns have remain clear for several months, with the largest risk coming from water droplets formed by condensation. At room temperature or 37°C, the plates eventually dry out. The patterns usually remain visible on dried out plates, especially after gently rinsing with water.

Susceptibility to variance in agar preparation: We have observed pattern formation with a wide range of agar percentages (0.15-0.35%) [12]. At percentages below 0.2% however, the agar falls apart easily. At percentages above 0.35%, the cells tend to not swarm. Furthermore, the pattern formation seems robust to small variations in LB concentration [28].

Assay sensitivity and specificity: The specificity of the assay is dependent on the nanobody portion of the adhesin. Nanobodies themselves can be highly specific and nanobodies (and ultimately their coding sequences) against virtually any target can be obtained commercially on the time scale of a few months [29, 30], affording this assay the potential to be highly specific as well. Furthermore, positive and negative controls can easily be implemented into the assay to validate the readout of the assay, improving reliability.

The sensitivity of such assays is likely in the range of micromolar concentrations as demonstrated here (Fig. 2d). However, higher sensitivities might be achievable with further optimization (which we did not attempt here), for example by varying the adhesion strength between cells using different expression levels or alternate nanobody clones [1]; furthermore, it may be possible to introduce amplification strategies by integrating higher level genetic circuits, or introducing secondary molecular amplifiers.

4 Text S4: Additional Experiments, Simulations, Data, and Analysis

4.1 Long range interaction artifacts present in transmutation pattern (Fig. 1d-f)

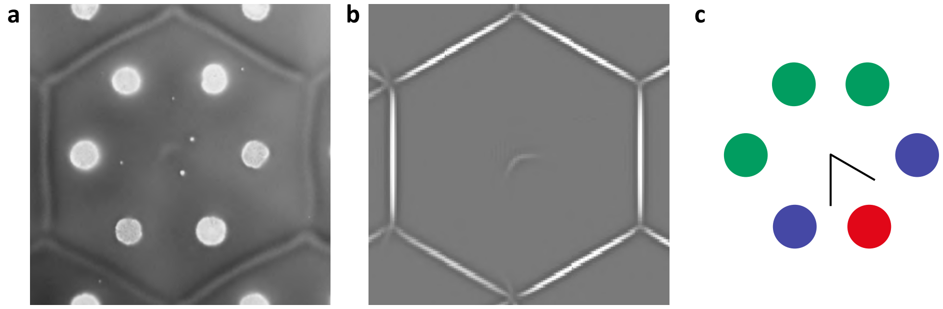

Figure S9: Faint and negligible long range patterning artifacts due to non-local interaction can be observed in both experiments and simulations in the transmutation pattern (Fig. 1d-f), which gives further credibility for the predictive power of the model. **(a)** Close up of experimental pattern shows a faint artifact in the center of hexagons. **(b)** This artifact can be detected more clearly in simulations. **(c)** This artifact (overemphasized by black lines) is due to long range interactions between Nb3 (green) and Ag3 (red) lines that become non-negligible at the six-fold vertices.

4.2 Magnified view of colorings

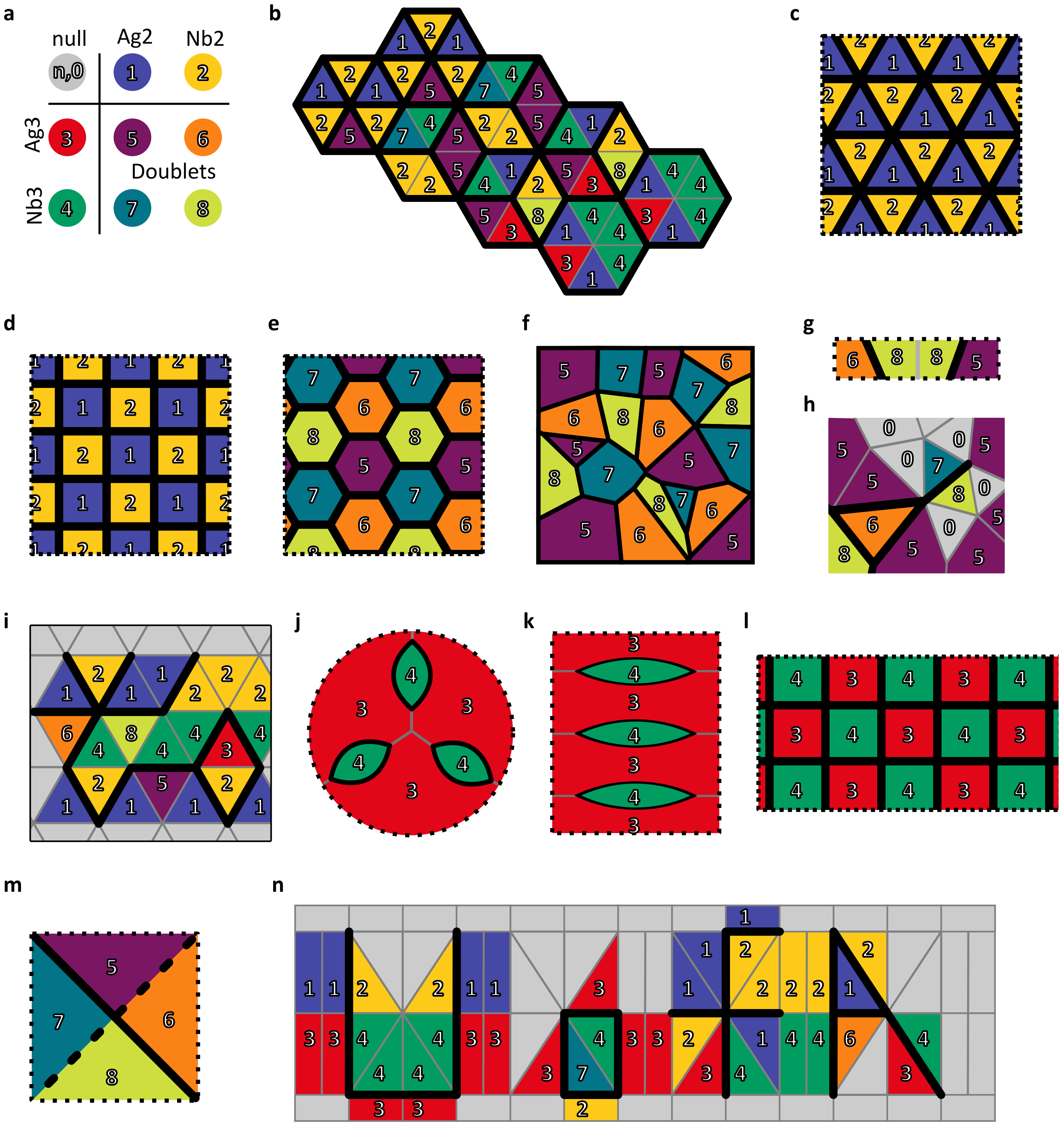

Figure S10: *(see next page)*

Figure S10 ***(previous page)*****:** Magnified view of colorings. **(a)** Coloring-adhesin map. Colorings for: **(b)** Escher-like pattern in Fig. 1d-f. **(c)** Regular triangular tiling in Fig. 4a (left). **(d)** Regular square tiling in Fig. 4a (middle). **(e)** Regular hexagonal tiling in Fig. 4a (right). **(f)** Random Voronoi pattern in Fig. 4b. **(g)** Schematic from Fig. 4c. **(h)** Definition of open, closed and hidden interfaces in Fig. 4d. **(i)** Triangular lattice algorithm Fig. 4e. **(j)** Curved interface pattern in Fig. 4f. **(k)** Cut-out pattern and wetting pattern in Fig. 4g, h. **(l)** Droplet-capture pattern in Fig. 4i. **(m)** Environmentally-dependent pattern in Fig. 4j. **(n)** 16-segment pattern in Fig. 4k. See main paper for direct visualization of formed and hidden interfaces.

4.3 Interface profiles and widths for various strain pairs

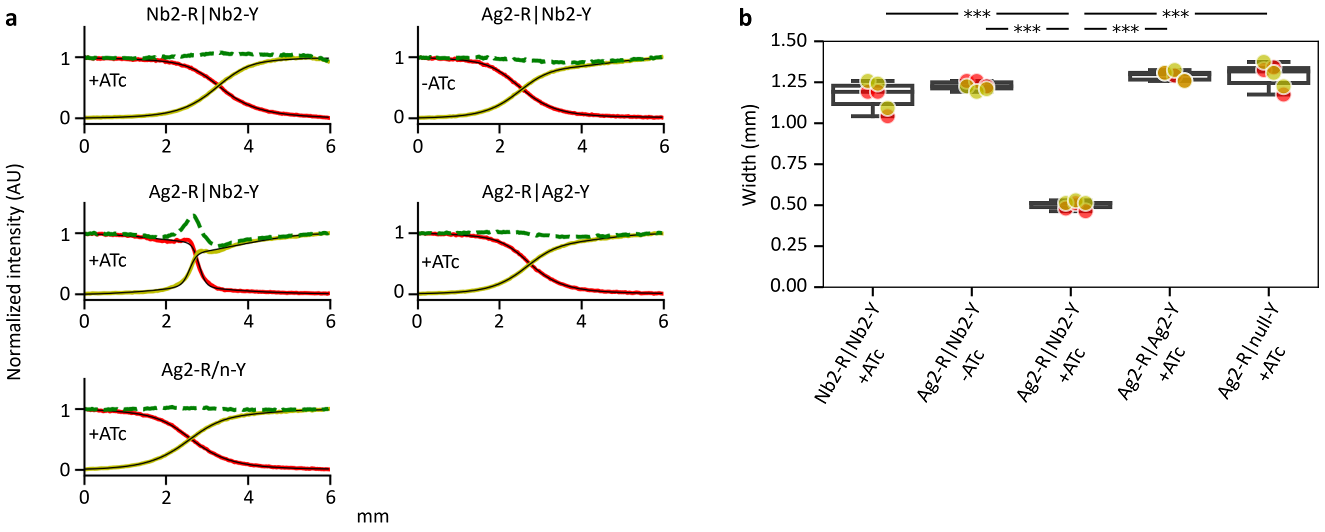

Figure S11: To control for confounding factors in Fig. 2a, we tested various experimental conditions and found that in all cases, sharp interfaces form if and only if cells express complementary adhesins. All experiments were analyzed as in Fig. 2a. **(a)** Representative interface profiles for various combinations of Ag2 and Nb2 pairs expressing YFP or RFP. Cells expressing non-complementary pairs (Nb2|Nb2, Ag2|Nb2, Ag2|n) with anhydrotetracycline induction (+ATc) form smooth interface profiles. Cells expressing complementary adhesins (Ag2|Nb2) but without induction (-ATc) also form smooth interface profiles. Cell expressing complementary adhesins (Ag2|Nb2) with induction form sharp profiles. **(b)** Quantification of interface width for triplicate samples represented in (A). The cumulative means for each experimental condition are 1.17±0.07 mm, 1.23±0.01 mm, 0.50±0.01 mm, 1.29±0.02 mm, and 1.29±0.07 mm. Errors are standard deviation, and (*, **, ***) indicate multiple comparison adjusted $p \leq 0.05, 0.01, 0.001$, respectively, calculated using Tukey's honestly significant difference test (HSD). Red and yellow colors correspond to RFP and YFP expressing cells respectively, dashed green lines correspond to cumulative cell density, and black lines correspond to fits. $n = 3$ replicates for all conditions. Box-plots label the median, quartiles, and extremes.

4.4 ATc induction sweep

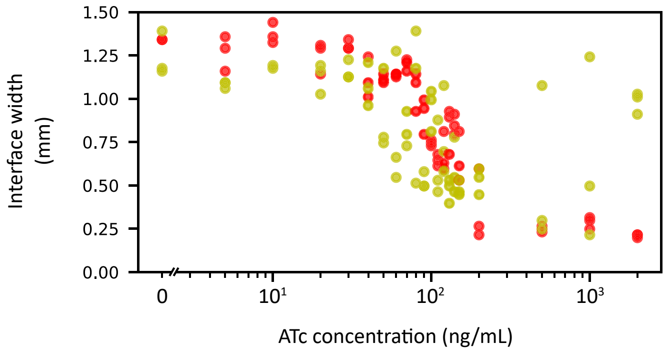

Figure S12: Interface widths for titrating anhydrotetracycline (ATc) sweep. Because adhesin expression is regulated by a TetR repressor, ATc concentration can be used to regulate adhesin expression levels [1]. In this experiment, we used the strains Ag2-RFP|Nb2-YFP, and varied the concentration of ATc over several orders of magnitude up to 2 $\mu g/ml$. By fitting the profiles to Eq. 1, we found the interface width is roughly 1.3 mm without ATc, and sharply decreases around 100 ng/mL ATc (the 1x concentration used throughout the paper). The interface width then plateaus to a minimum of roughly 0.4 mm starting roughly at 200 ng/mL ATc, with deviating behavior beyond that. We note that the TetR response function is likely non-linear at extremely low and high ATc concentrations. Consequently, in the main paper we kept the induction level constant at 100 ng/mL ATc and employed competitive peptide inhibitors, whose inhibitory effects has a much clearer physical interpretation. Red and yellow dots correspond to Ag2-RFP and Nb2-YFP cell respectively. $n = 3$ replicates for each concentration.

4.5 Serial dilution leads to linear delay in swarming

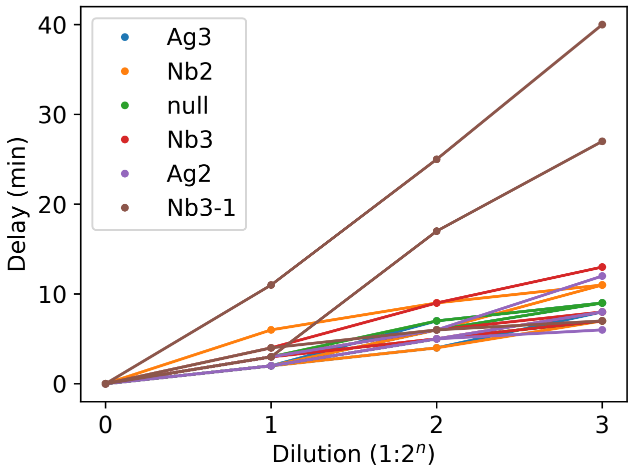

Figure S13: Serial dilution of initial cell seeding concentration leads to linear increase in delay for swarms to reach 8mm relative to full concentration seeding. This delay is attributable to a delay in the initiation of swarming since initial seeding concentration has no effect on eventual expansion rates. Data shown are individual experiments from Fig. S14a. Note that Nb3-1 has on average a much longer delay (and also appears more variable), while all other strains show very similar behavior. $n = 3$ replicates for Ag2, Nb2, Ag3, and Nb3-1. $n = 4$ replicates for null and Nb3.

4.6 Growth and expansion rate measurements

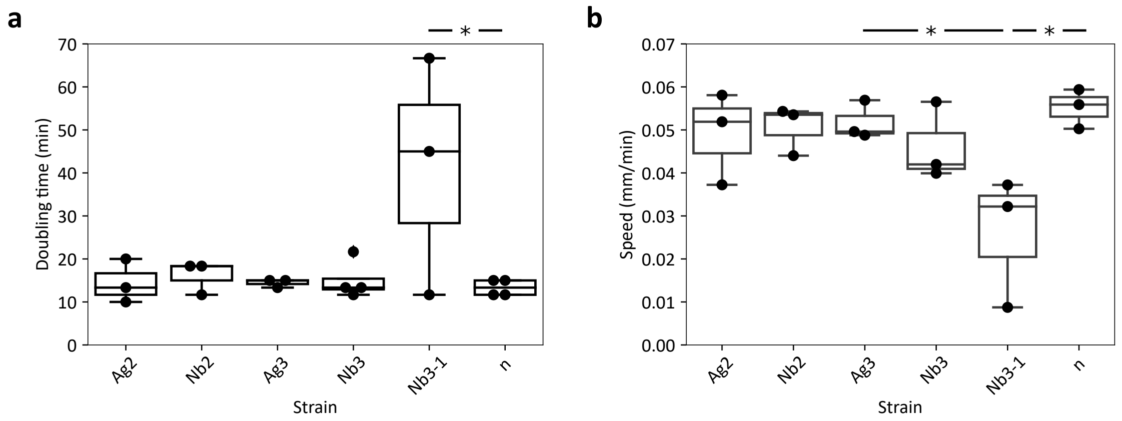

Figure S14: Cell doubling, and swarm expansion rate measurements. **(a)** Cell doubling time for various strains. We report the mean doubling times for Ag2 as 14.4 ± 4.2, for Nb2 as 16.1 ± 3.1 min, for Ag3 as 14.4 ± 0.8 min, for Nb3 as 15.0 ± 3.9 min, for Nb3-1 as 41.1 ± 22.6 min, and for null as 13.3 ± 1.7 min. The average doubling time for all strains excluding Nb3-1 was 14.6 ± 3.2 min. $n = 3$ replicates for Ag2, Nb2, Ag3, and Nb3-1. $n = 4$ replicates for null and Nb3. **(b)** Swarm expansion rates for various strains. We report the mean expansion rates for Ag2 as 0.0491 ± 0.0087 mm/min, for Nb2 as 0.0506 ± 0.0047 mm/min, for Ag3 as 0.0518 ± 0.0037 mm/min, for Nb3 as 0.0461 ± 0.0074 mm/min, for Nb3-1 as 0.0260 ± 0.0124 mm/min, and for null as 0.0552 ± 0.0038 mm/min. The average expansion rate for all strains excluding Nb3-1 was 0.0506 ± 0.0067 mm/min. Errors are reported as standard deviation, and (*) indicates multiple comparison adjusted $p < 0.05$ calculated using Tukey's HSD. Standard box-and-whisker plots report median, quartiles and extremes. $n = 3$ replicates for all strains. Box plots label the median, quartiles, and extremes. Methods: Overnight cultures were seeded on 0.225 % (w/v) soft agar with antibiotics and ATc, placed in a 37°C incubator, and imaged overnight using a custom Raspberry Pi imaging platform. Frames were captured every 5 minutes. To measure cell doubling times, we seeded four 1:2 serial dilution of overnight cultures of cells expressing adhesins. Taking images every five minutes, we measured the delay to reach an 8 mm radius. The doubling time was calculated by dividing the total time delay by the number of 1:2 serial dilutions. To measure migration speed, the radius of the chemotactic front was measured using ImageJ. The migration speed was calculated from the slope using linear regression.

4.7 Seeding initial conditions beyond point sources

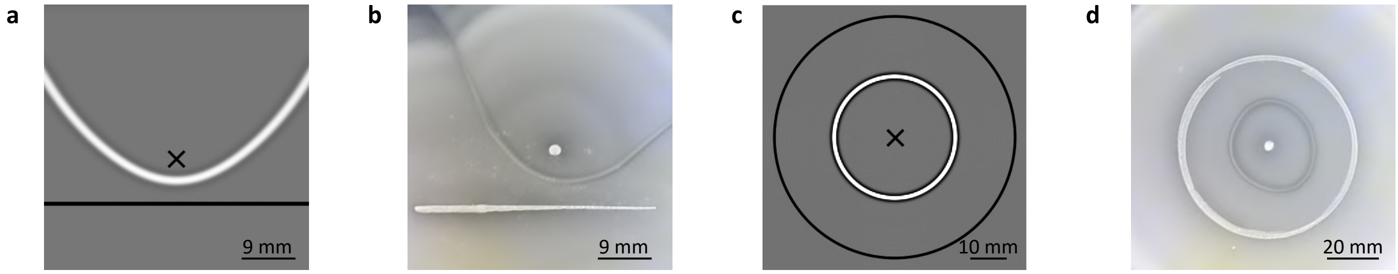

Figure S15: Non-point source initial seeding conditions behave like the summation of many points. **(a)** Simulation and **(b)** experimental realization of interface pattern formed between a line (Ag2) and point seeding (Nb2), approximating the mathematically expected parabola. **(c)** Simulation and **(d)** experimental realization of interface pattern formed between a circle (Ag2) and point (Nb2) seeding, approximating the expected circle. For simulations, black points and lines are initial seeding conditions and white lines are the formed interfaces. See supplementary text S1 for modeling details. See also Fig. 2h.

4.8 Confocal image segmentation

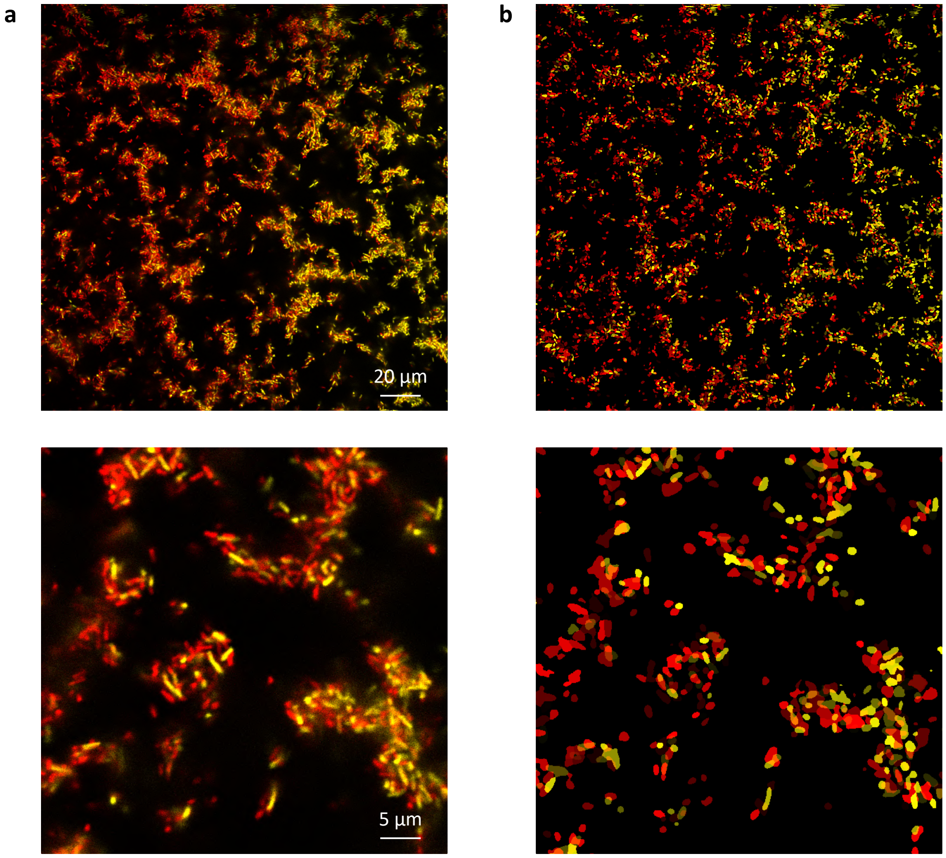

Figure S16: Confocal images were segmented using DeepCell, a machine learning cell segmentation library for Python [31]. **(a)** Confocal images of Ag2-RFP|Nb2-YFP (top) with magnified image (bottom). **(b)** DeepCell segmentation of corresponding image, with different shades assigned to uniquely identified cells.

4.9 Fluorescence intensity vs. cell counts

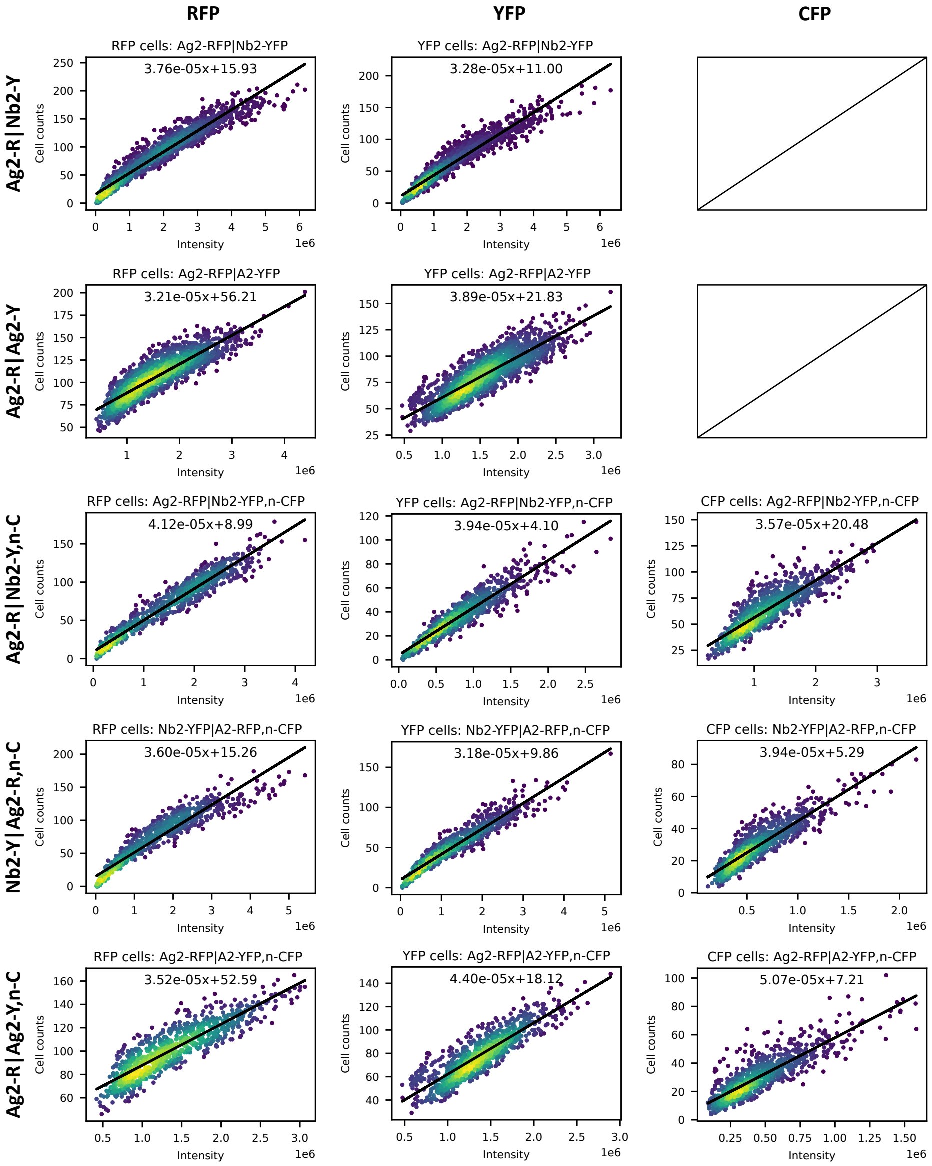

Figure S17: *(see next page)*

Figure S17 ***(previous page)*****:** To validate the use of fluorescence intensity as a proxy for cell counts in our analysis (Figs. 2, 3), plots of cell counts as a function of fluorescence intensity in confocal images for various fluorophore/adhesin combination were plotted, and reasonable correlation was found. Each point corresponds to a slice of a confocal image frame. Cell counts were determined by DeepCell segmentation as shown in figure S16. Paired confocal and segmentation images were broken up into 128 pixel wide bins. Intensities are arbitrary units. Equations immediately above plots are fit functions. Data from several images were combined, and fitted using linear regression.

4.10 Confocal analysis of transition region

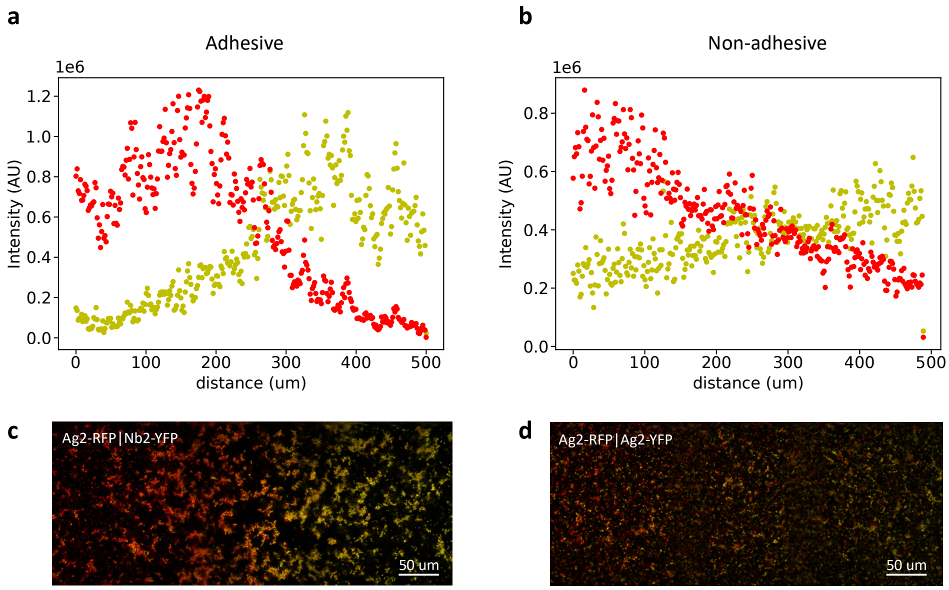

Figure S18: Interface profiles measured from confocal images recapitulate epifluorescence profiles. To validate that confocal images recapitulate the macroscopic interface profiles (Fig. 2a/c, 3a/b), we measured the interface profiles in confocal images. First, stitched confocal images spanning the interface region were binned into 16 pixel width strips, and the fluorescence intensity was measured and plotted as a function of position. Confocal images show a transition region on the same order ($\sim 400\mu m$) as the macroscopic images for adhesive conditions. **(a)** Interface profile for adhesive Ag2-RFP|Nb2-YFP interface, and **(b)** non-adhesive Ag2-RFP|Ag2-YFP interface. **(c)** Confocal images for adhesive (a) and **(d)** non-adhesive (b) respectively.

4.11 Fitting pair correlation functions

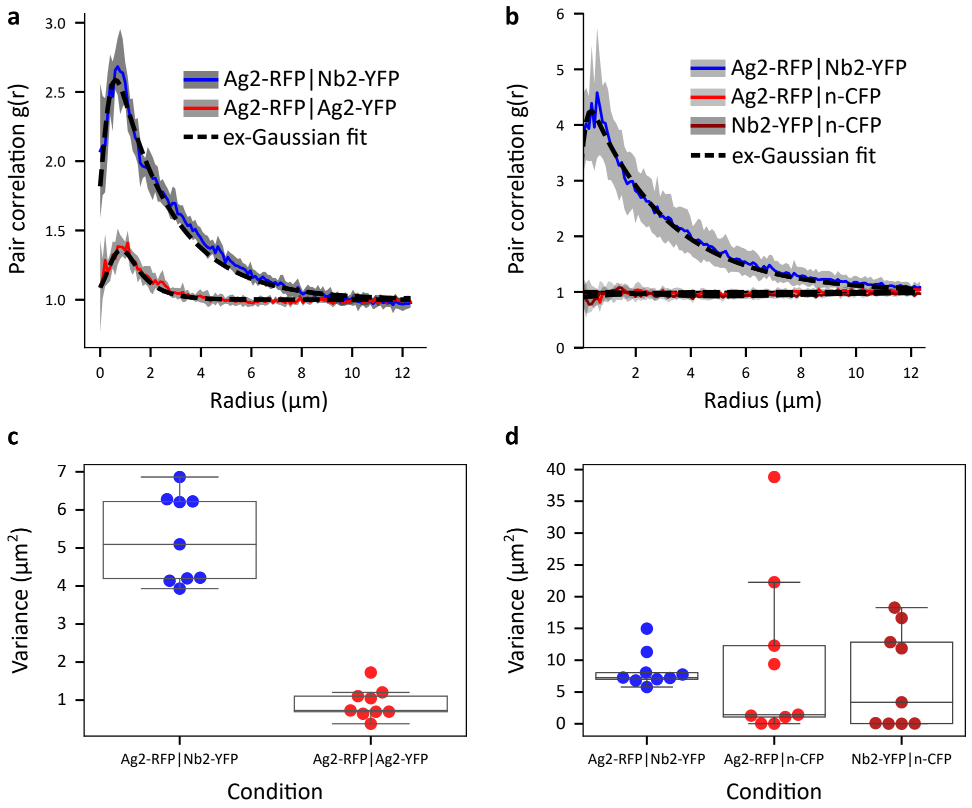

Figure S19: *(see next page)*

Figure S19 ***(previous page)*****:** The width of the first peak in the pair-correlation function (Figs. 3a, b) reflects the characteristic cluster size within an image. Exponentially modified Gaussian ('ex-Gaussian') fits to the first 12 μm of the pair-correlation function in Fig. 3a used the following function: $f(x;\mu,\sigma,\lambda) = \frac{\lambda}{2}e^{\frac{\lambda}{2}(2\mu+\lambda\sigma^2-2x)}\,\mathrm{erfc}\,(\frac{\mu+\lambda+\sigma^2-x}{\sqrt{2}\sigma})$. To fit the ex-Gaussian, we first converted the pair-correlation function to a probability density function by subtracting one and normalizing by the area under the curve. To plot the ex-Gaussian alongside the pair-correlation function, we reversed this conversion by multiplying by the area under the curve and adding 1. We calculated the variance of the ex-Gaussian distribution with: $\mathrm{Var}\,(\sigma,\lambda) = \sigma^2 + \frac{1}{\lambda^2}$ **(a)** For the averaged pair correlation function for the adhesive (Ag2-RFP|Nb2-YFP) pair, we report an ex-Gaussian variance of $5.0330\mu\mathrm{m}^2$, and for the non-adhesive pair (Ag2-RFP|Ag2-YFP) an ex-Gaussian variance of $0.8089\mu\mathrm{m}^2$. **(b)** This analysis was also applied to images corresponding to Fig. 3b. For the averaged pair correlation function for the adhesive (Ag2-RFP|Nb2-YFP) pair we report an ex-Gaussian variance of $8.2818\mu\mathrm{m}^2$. The non-adhesive pairs did not have an appreciable peak, and therefore had inflated ex-Gaussian variances of $200\mu\mathrm{m}^2$ (Ag2-RFP|n-CFP) and $19.3\mu\mathrm{m}^2$ (Nb2-YFP|n-CFP). Shaded regions are standard deviation for $n = 9$ experiments. **(c)** Ex-Gaussians were fitted to individual replicates (n=9) for experiment (a), and the variance was calculated. For the adhesive condition, the average variance was $5.24 \pm 1.09\mu\mathrm{m}^2$, and for the non-adhesive condition the average variance was $0.91 \pm 0.38\mu\mathrm{m}^2$. An independent two-tailed t-test reports a significance of $p = 10^{-8}$. **(d)** Ex-Gaussians were fitted to individual replicates (n=9) for experiment (b), and the variance was calculated. For the adhesive (Ag2-RFP|Nb2-YFP) pair, the average variance was $8.46 \pm 2.71\mu\mathrm{m}^2$. For the non-adhesive (Ag2-RFP|n-CFP) pair the average variance was $9.62 \pm 12.53\mu\mathrm{m}^2$, and for the non-adhesive (Nb2-YFP|n-CFP) pair the average variance was $7.02 \pm 7.34\mu\mathrm{m}^2$. An independent two-tailed t-test reports a significance of $p = 0.80$, 0.52, and 0.51 respectively. Ex-Gaussian fits of non-adhesive conditions in (b) seem to have a bimodal distribution, resulting from poor fits. Note these results cannot be directly compared to those in Fig. S24, as one measures variance while the other directly measures cluster size. Furthermore, the cluster area quantification overemphasizes the contribution of single cells and small clusters, due to the fact that each cluster is weighed equally. Nevertheless, the quantification are of similar magnitude (cluster area of $\sim 7\ \mu\mathrm{m}^2$ vs. variance of $\sim 5\ \mu\mathrm{m}^2$), suggesting overall agreement.

4.12 Confocal stacks of interface region reveals 3-dimensional structure

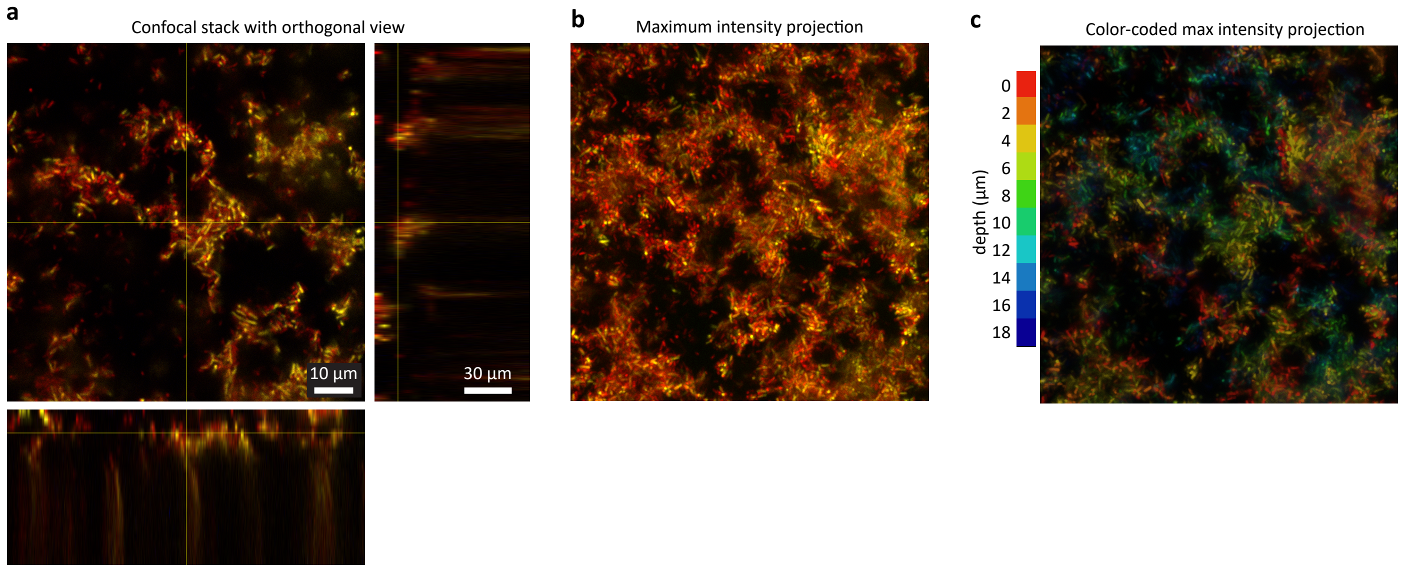

Figure S20: Confocal fluorescence microscopy z-stacks reveal a highly interconnected, 3-dimensional network of cells. **(a)** Confocal stack shown with orthogonal views taken at 2 μm sections of adhesive Ag2-RFP|Nb2-YFP cells at an interface. In the orthogonal views, the smearing of the images is due to displacement of the soft agar when attempting to image at greater depths. **(b)** Maximum projection intensity images of the first ten slices, corresponding to 20 μm total, suggests a highly connected network of cells. **(c)** Color coding the z-slices by depth verifies that the cells at differing heights are locally connected to the immediately adjacent stacks. (See also Fig. S21 for further characterization of the 3-dimensional structure.)

4.13 Extracting patterned bacterial-agar sheets; characterizing z-profiles of interfaces

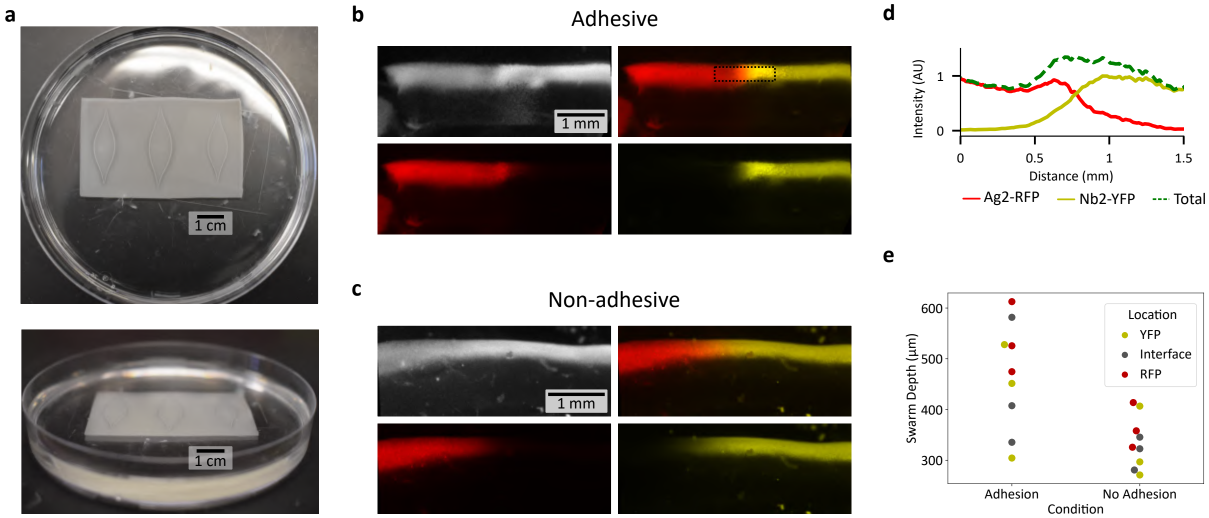

Figure S21: *(see next page)*

Figure S21 ***(previous page)*****:** Patterned bacterial-agar sheets form materials that can be cut-out, handled and imaged. Imaging reveals that these swarming bacteria deeply colonize soft agar, with complete penetration of cells at the adhesive interface. (In contrast, we note that well defined monolayers of swarming *E. coli* have only been reported previously with very specific agar formulations [32].) Plates were grown with antibiotics and ATc at 37°C. **(a)** We were able to cut-out bacteria-agar sheets that contained interface patterns. (The seeding pattern for the specific interface pattern shown here is taken from Fig. 4g.) These sheets can be cut into desired shapes, and then handled further in liquid (e.g., PBS) or on top of hard surfaces. These sheets are fragile and have to be handled with care. Top-down (top) and isometric view (bottom) of Petri dish with cut-out agar. **(b)** A cut-out sheet placed on its side was imaged with oblique illumination (grey) and wide-field epifluorescence (red, yellow) microscopy (Leica M205 fluorescent microscope). This lateral view reveals that bacterial swarms (Nb3-RFP|Ag3-YFP) colonize approximately 0.47mm into the surface of the soft agar. Furthermore, the area immediately below the interface reveals a relatively faint strand of clustered cells that seem to span further down (i.e., the full height of the gel) under oblique illumination. However, no fluorescence signal is detectable in these deeper clusters. **(c)** Non-adhesive controls (Nb3-RFP|Ag2-YFP) colonize approximately 0.34mm into the surface, but lack the strand at the interface region visible under oblique illumination that spans the full height of the agar. We leave the biophysical characterization of how these deep interface strands form for future work. **(d)** Measuring spatial variation of the fluorescence intensity in the adhesive condition (boxed region in (b) - upper right panel) reveals a peak in fluorescence at the interface, verifying that the increase in fluorescence intensity is attributable to an increase in cell density as opposed to a build-up of cells in the vertical direction (in agreement with Fig. 2a). **(e)** Measuring swarm depth with and without adhesion at different positions (i.e., at the interface and on either side of the interface) suggests that strains expand vertically to differing extents within and between experiments. However, statistical tests did not reveal a significant difference. Cell swarms in adhesive conditions on average were 469±83μm deep, while in non-adhesive conditions they were 336±28μm deep (p-value = 0.098 using an independent T-test). Comparing RFP and YFP expressing strains within the adhesive condition reveals RFP-expressing swarms were on average 452±98μm deep, while YFP-expressing swarms were 376±93μm deep (p-value = 0.069 using a paired T-test). All other comparisons were also insignificant. Errors are standard deviation. At the interface region, the swarm depth was measured up the extent of the fluorescent region for the adhesive cases. $n = 3$ replicates for all conditions.

4.14 Measurements of surface topology suggest a smooth interface profile

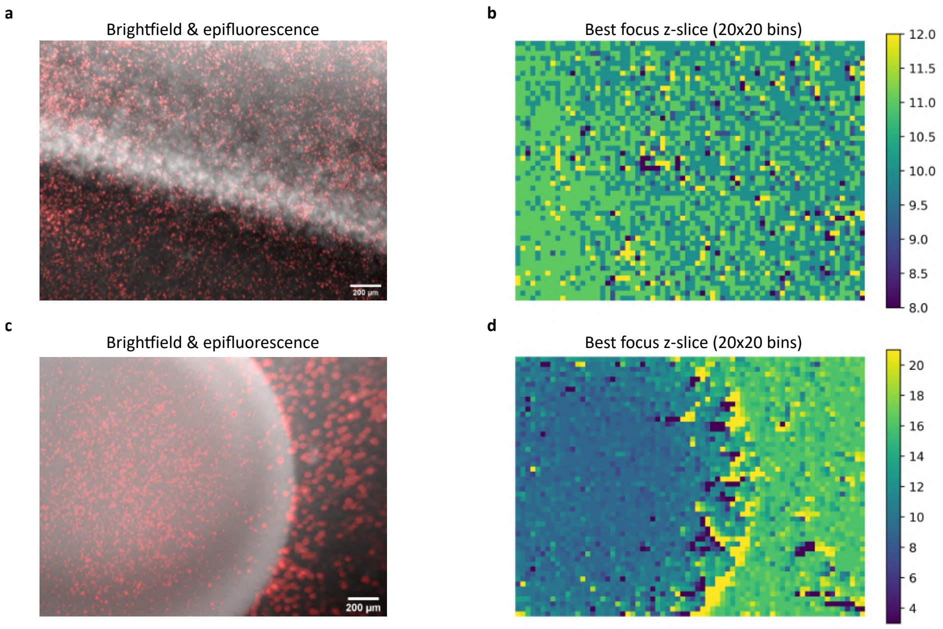

Figure S22: We did not observe any significant changes in surface topology at the interface, while the site of inoculation substantially rises above the rest of the plane. **(a)** To determine whether the interfaces lead to changes in surface topology of the agar, carboxylated fluorescent beads (2 μm, Red 580/605, Molecular Probes Fluorospheres F8826) were placed on the surface of the soft agar in which Ag3 and Nb3 swarms had formed an interface after growth at room temperature. Room temperature conditions were used because it produced the strongest interfaces with this strain combination. Experiments at 37°C produced similar results. Z-stacks of step size 10 μm were acquired with oblique illumination (grey) and epifluorescent (red) microscopy (Leica M205 fluorescent microscope). A single slice is shown here. The interface corresponds to the bright grey stripe running diagonally from the upper left to the lower right. **(b)** To localize the z-position of the beads, the images were tiled into bins of 20x20 pixels, and the Laplacian of the Gaussian was used to define the z-slice with best focus. These measurements reveal a systematic change in height diagonally across the the image of approximately 10 μm which we attribute to an uneven imaging surface. Nonetheless, we do not observe any systematic fluctuations in the agar height across the interface. These results indicate that any potential changes in surface height due to adhesins are smaller than 10 μm. **(c)** The inoculation site was also imaged, using the same conditions as described in (a). **(d)** Measuring the z-position of the inoculation site reveals the inoculation site is tens of microns higher than the rest of the agar, which is consistent with visual observations, and serves as a positive control for this analysis. The color bars are in units of z-steps, where each z-step is 10 μm. With small bins, some subtiles lack any beads resulting in noisy measurements. To minimize this artifact, we clipped the measurements to a range of $\pm$ 2 standard deviation around the mean. Three independent experiments produced similar results.

4.15 Pair correlation analysis in single images

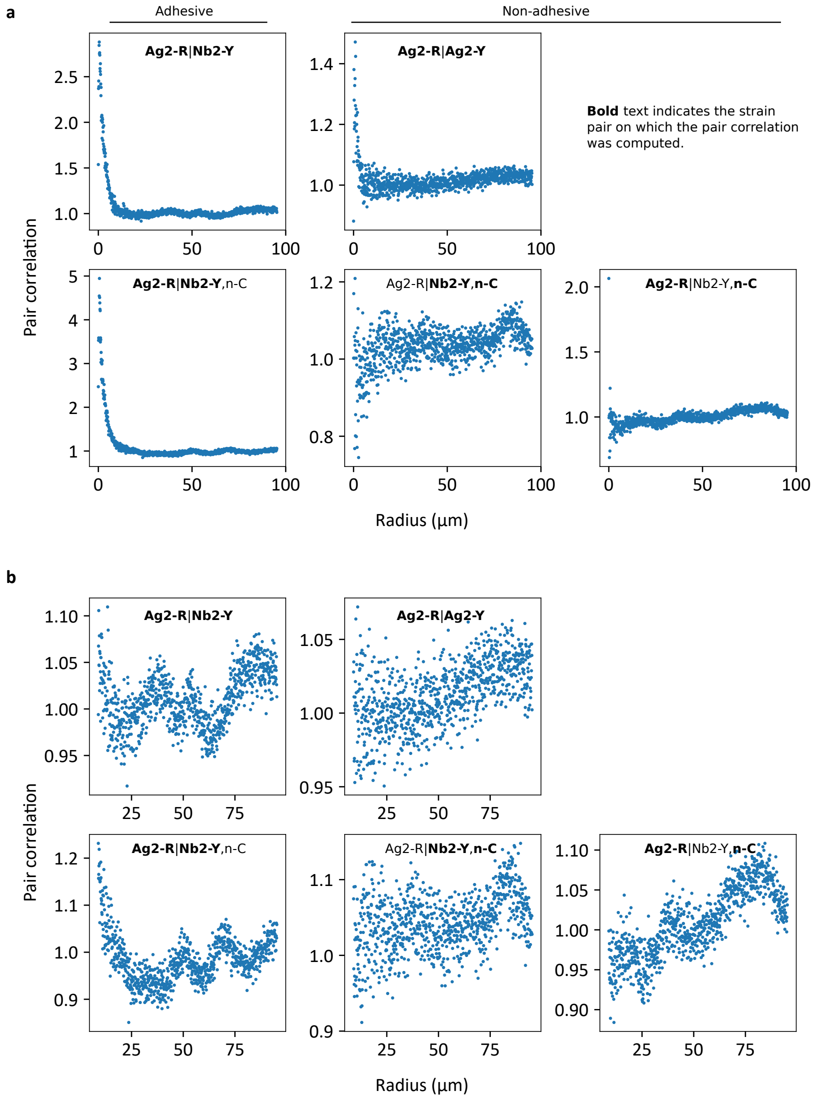

Figure S23: Pair-correlation function for individual example experiments without averaging reveals a periodicity that corresponds to inter-cluster spacing. This type of information is washed out when averaging multiple experiments as in figures 3a,b or S19. **(a)** Zoomed-out mixed species pair-correlation function for individual experiments. Bold inset text denotes the strains for which the pair-correlation function has been computed. Adhesive conditions lead to an increase in width for the first peak, which scales with the characteristic radii of the adhesive clusters. **(b)** Zoomed-in view of (a), showing a periodicity to the pair correlation function in experiments with cluster formation (all expect Ag2-R|Ag2-Y). This periodicity has a characteristic spacing of roughly 25μm, which likely corresponds to center-to-center intercluster spacing.

4.16 Confocal analysis of cluster area and mean free path

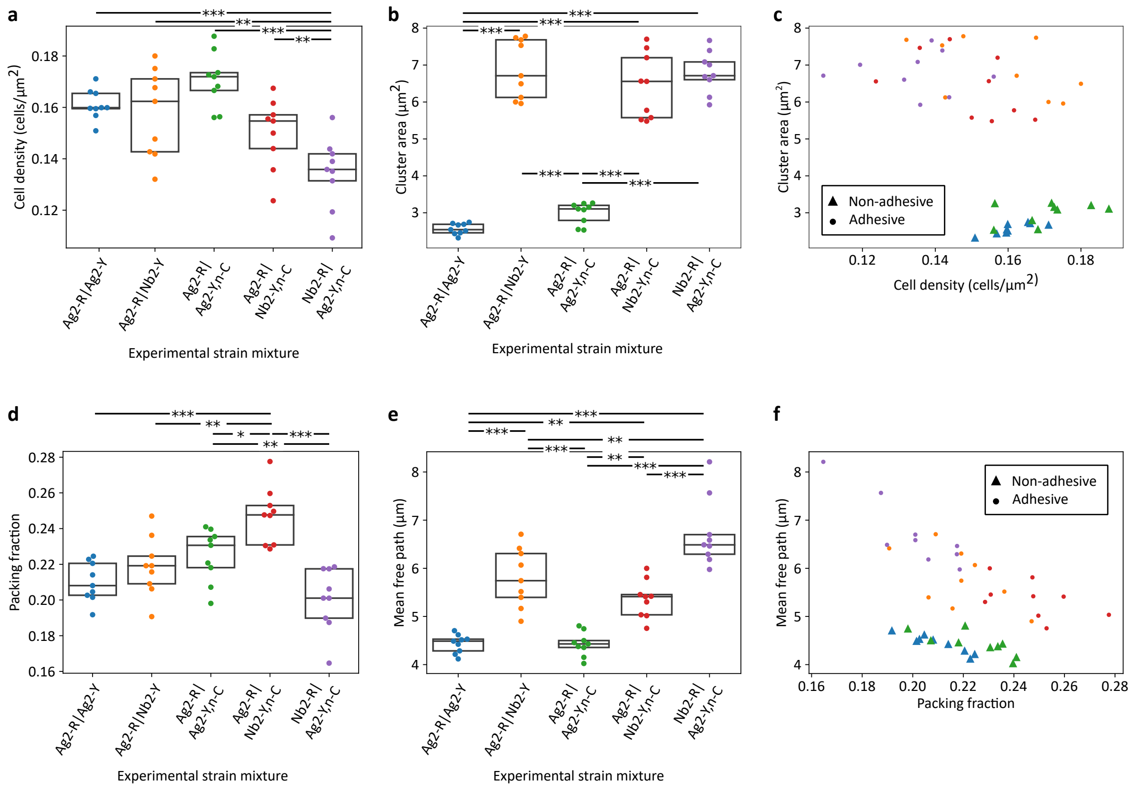

Figure S24: *(see next page)*

Figure S24 ***(previous page)*****:** Analysis of confocal images of the interface regions for various experimental conditions shows increase in cluster area and mean free path for adhesive cells. All quantification were performed on images segmented using DeepCell. Overall, this analysis shows an increase in cluster area and mean free path for adhesive cells. **(a)** Average cell density calculated for each image shows no systematic trend for adhesive vs non-adhesive experiments. The cumulative means for each experimental condition are 0.161±0.006 cells/μm^2, 0.158±0.016 cells/μm^2, 0.171±0.010 cells/μm^2, 0.150±0.013 cells/μm^2, and 0.135±0.013 cells/μm^2. Average cell density for all experimental conditions is 0.155±0.012 cells/μm^2. **(b)** Average cluster area calculated for each image shows systematic increase in cluster area for adhesive cells. The cumulative means for each experimental condition are 2.56±0.14 μm^2, 6.89±0.74 μm^2, 2.99±0.27 μm^2, 6.43±0.83 μm^2, and 6.80±0.53 μm^2. **(c)** Plotting cell density vs. cluster area to control for interdependence of these variables shows that non-adhesive conditions clearly separate out from adhesive experimental conditions. **(d)** Packing fraction calculated for each image as the sum of the segmented cell pixels over the sum of all the pixels in the image also did not reveal a clear between adhesive and non-adhesive conditions. The cumulative means for each experimental condition are 0.210±0.011, 0.217±0.016, 0.225±0.014, 0.247±0.015, and 0.200±0.017. **(e)** Mean-free path perpendicular to the interface calculated for each image shows that interfaces have a systematically increased free path. Mean free path length was computed as average length of contiguous non-occupied pixels in a segmented image, in the direction perpendicular to the interface using a custom Python script. The cumulative means for each experimental condition are 4.43±0.18 μm, 5.80±0.58 μm, 4.43±0.23 μm, 5.36±0.37 μm, and 6.72±0.67 μm. **(f)** Plotting packing fraction vs. mean free path to control for their interdependence shows that although there is a clear relationship between packing fraction and mean free path length, non-adhesive conditions cluster separately from adhesive experimental conditions, and hence the mean-free path with adhesion cannot be simply explained by changes in packing fraction. Errors are standard deviation, and (*, **, ***) indicate multiple comparison adjusted $p \leq 0.05, 0.01, 0.001$ calculated using Tukey's HSD. $n = 9$ replicates for all conditions. Box plots label the mean and quartiles.

4.17 Selective interface experiment combinations

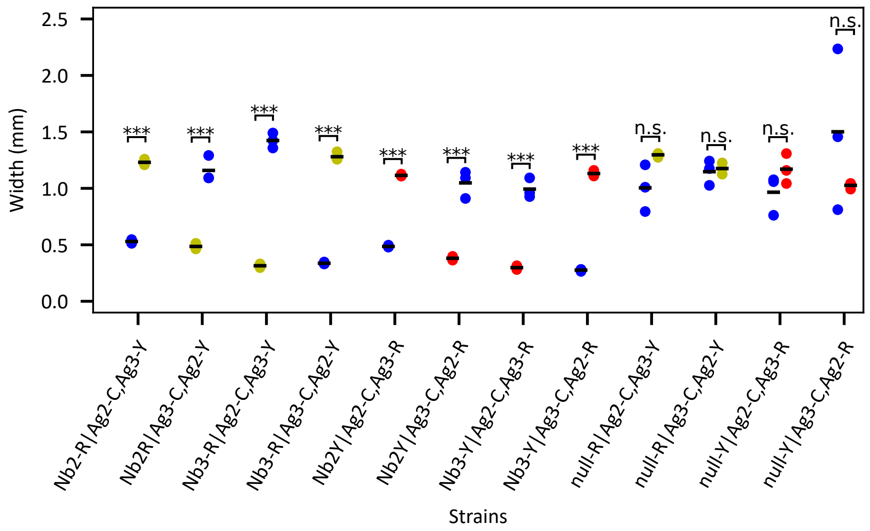

Figure S25: Discriminating between cell types at the interfaces enables encoding of complex behavior between swarming cells. To demonstrate selectivity of the interface, we tested various combinations of cell types. In these experiments, cells were seeded in two separate positions 9 mm apart. In one position, there was only a single strain. In the other, there was a mixture of two strains. All three strains express distinct fluorophores. We measured the interface profiles using fluorescent microscopy (see Eq. 1), and found that within the two strain mixture, the strain expressing the complementary pair to the single strain seeding consistently formed sharper interfaces than the non-complementary strain. Note that also various other combinations between null, singlet, and doublet strains were tested through various experiments, e.g., in figures 1f, 4e,h, which also always led to the expected results. Various selective interface profiles shows adhesive strain always forms narrower interface in mixed-species experiments. Complementary strain (when applicable) is always the leftmost of the pair. Note, the unmixed strain is not included in these plots. Triplicates plotted for each condition. Means plotted with a horizontal bar. Colors correspond to the fluorophore/adhesin combination. (*, **, ***) indicate $p \leq 0.05, 0.01, 0.001$ and were calculated using an independent T-test for each pair. $n = 3$ replicates for all conditions.

4.18 Simulation and experimentation of all three regular periodic tilings

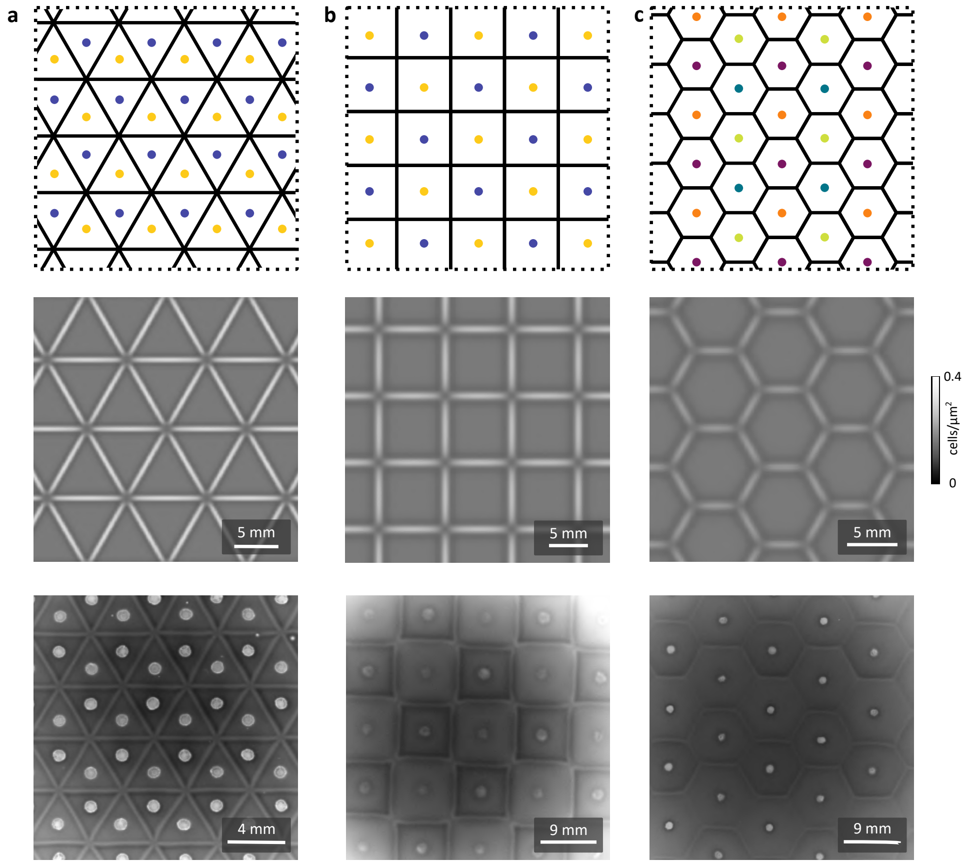

Figure S26: Seed (top), simulation (middle) and experimental realization (bottom) of all three regular periodic tilings. This includes **(a)** triangular, **(b)** square **(c)** and hexagonal tilings.

4.19 Additional simulation results

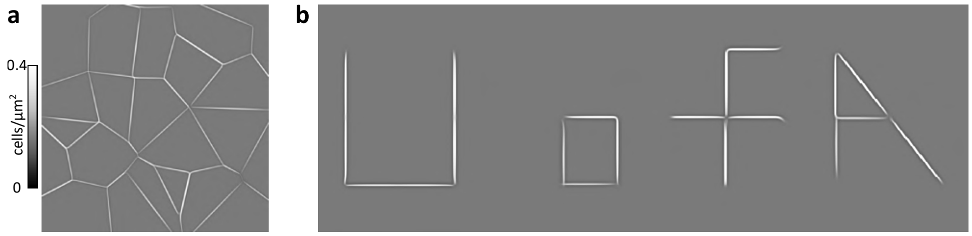

Figure S27: Simulation results for **(a)** random Voronoi tiling and **(b)** 16-segment display (see Fig. 4).

4.20 Extensibility of bits

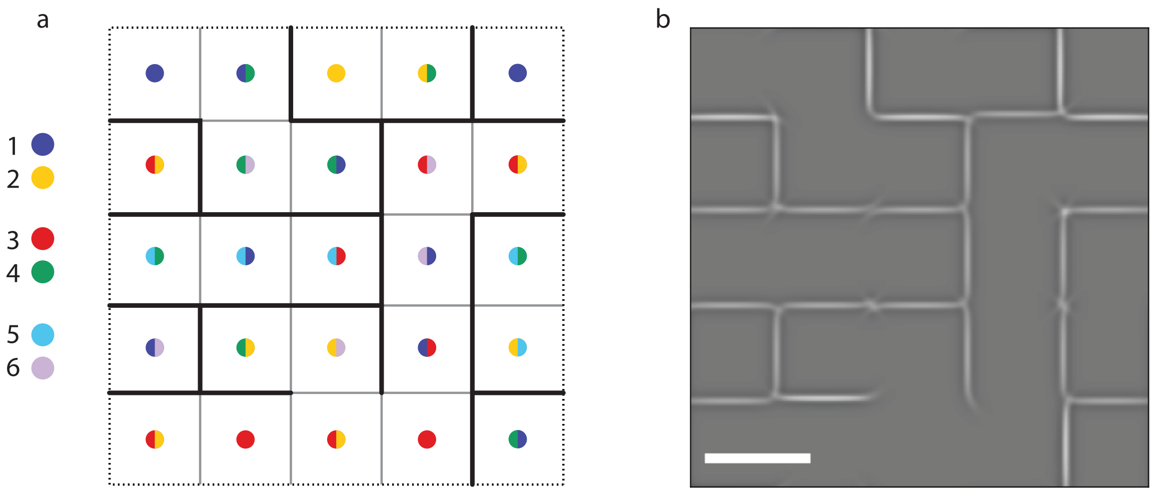

Figure S28: Simulation demonstrating the use of 3 adhesin pairs to create an arbitrary design on a square lattice. **(a)** Seeding pattern of a square lattice with target interface pattern overlaid. To realize this pattern, we use 3 adhesin pairs chosen by applying the algorithm from Fig. S8, which results in every seeding point containing at most two out of a possible six strains. **(b)** Simulation results at time $t = 120$ min from the seeding pattern in (a) demonstrating the target pattern. Scale bar is 5mm.

4.21 Patterned surface wetting properties

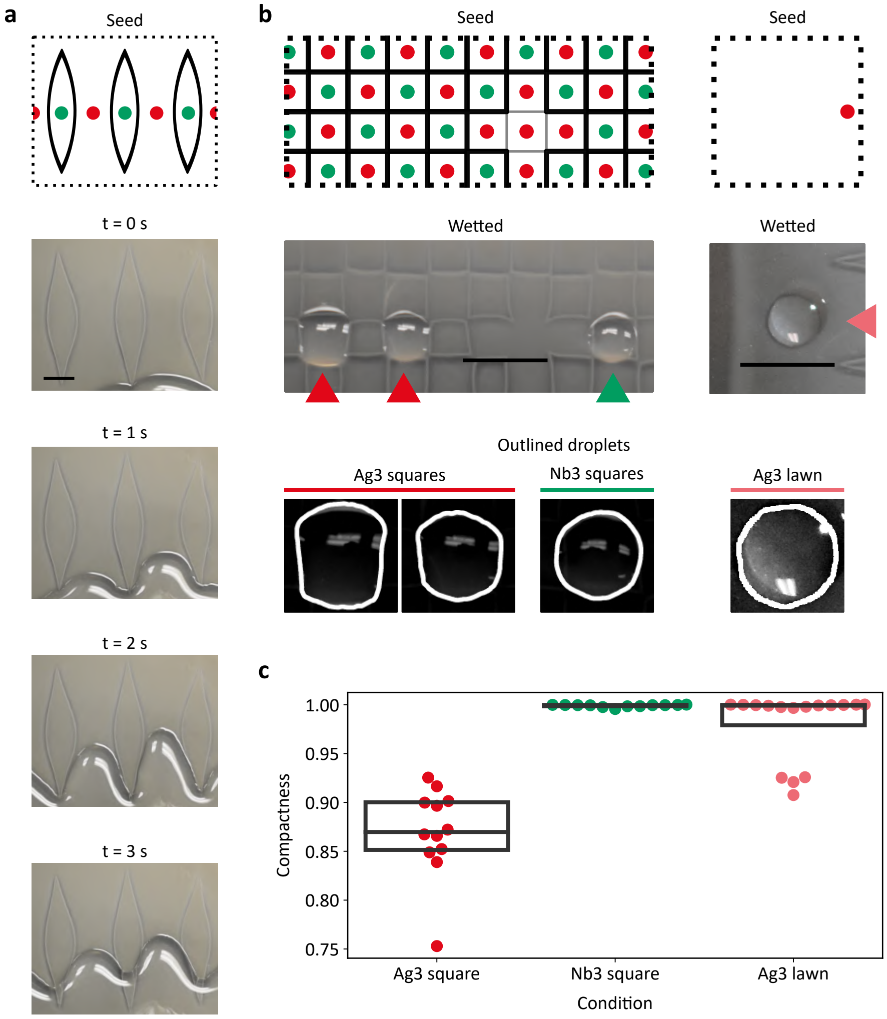

Figure S29: *(see next page)*

Figure S29 ***(previous page)*:** Surface wetting properties can be controlled using adhesin mediated soft-agar patterning. All plates were grown at room temperature (RT) for 48h, which produced sharper Nb3|Ag3 interfaces than at 37°C and seems to make wetting effects more robust. (Note: When grown at RT, Nb3 and Ag3 cells often do not leave a high density mark at the seeding position as seen at 37°C). **(a)** Generating an alternating Nb3 and Ag3 pattern with curved interfaces (top) and then pouring a liquid (1x PBS) onto the agar reveals that liquid flow is strongly affected by the patterning (image sequence), indicating that the wetting properties of the agar surface are locally changed in correlation with the formed Nb3|Ag3 interfaces (Video S10). Experiments with Nb2|Ag2 interfaces showed a similar but weaker effect. Control experiments with an equivalent pattern using Nb2|Ag3 or Nb3|Ag2 interfaces neither produced visible interfaces nor changes in wettability, i.e., the liquid just flowed across these invisible interfaces unhindered. Experiments were repeated at least three times. **(b)** To explore spatially constrained liquid droplets using these interfaces, we patterned checkerboard-like grids of alternating Ag3 and Nb3 squares and pipetted $10\mu l$ PBS droplets onto either squares. Droplet morphologies differed depending on the strain: Droplets on Ag3 squares (red arrow head) were angular and aligned with the shape of the underlying grid, while droplets on Nb3 squares (green arrow head) were more compact and round. Droplets on a lawn of Ag3 cells (light red arrow head) far from any interfaces also produced rounder droplets - in contrast to those on Ag3 squares. **(c)** To quantify these differences in droplet shape, we measured their compactness, which is defined as $4\pi A/p^2$, with droplet area, A, and perimeter, p. A and p were measured by tracing droplets in ImageJ FIJI. A circle and square have a compactness of 1 and $\pi/4$, respectively. Compactness measurements show that droplets on Nb3 squares and Ag3 lawns are significantly rounder than Ag3 squares ($p < 0.001$ for both, independent t-test). This shows that interface patterns can affect droplet shapes, but depends on the specific adhesins, i.e., the presence of a visible interface by itself is not sufficient for restricting liquid flow. Conversely, the round droplet morphology on the Ag3 lawn shows that the material property of the substrate by itself is insufficient to restrict liquid flow. A possible mechanism consistent with these observations is that Ag3 strains create a more hydrophilic surface, while Nb3 strains create a more hydrophobic surface, and a sharp interface between the strains is necessary to create sharp gradients in hydrophobicity to contain droplets. However, we cannot rule out the possibility that the interfaces themselves have inhomogenous properties. This is not a comprehensive analysis of this wetting phenomena, which is left to future work. However, it demonstrates the ability to pattern surface wetting properties, which could have useful applications in generating novel biomaterials, or studying biofilm surface properties [33]. The non-circular droplets in squares were not achieved in every experiment, and were dependent on the formation of strong interfaces. Plates grown at 37°C did not produce as strong of a wetting effect, which may be correlated with the strength of the interface formed. We observed the square like droplets inside an Ag3 grid on two independent days (7 and 5 square-like droplets each). In contrast, we never observed square-like droplets on Nb3 grids (2 and 10 droplets) or on Ag3 lawns far from interfaces (16 droplets). Scale bars are 1cm.

References

1. Glass, D. S. & Riedel-Kruse, I. H. A synthetic bacterial cell-cell adhesion toolbox for programming multicellular morphologies and patterns. *Cell* **174,** 649–658 (2018).

2. Cremer, J. *et al.* Chemotaxis as a navigation strategy to boost range expansion. *Nature* **575,** 658–663 (2019).

3. Budrene, E. O. & Berg, H. C. Complex patterns formed by motile cells of Escherichia coli. *Nature* **349,** 630–633 (1991).

4. Ben-Jacob, E., Cohen, I. & Levine, H. Cooperative self-organization of microorganisms. *Advances in Physics* **49,** 395–554 (2000).

5. Burns, K. J., Vasil, G. M., Oishi, J. S., Lecoanet, D. & Brown, B. P. Dedalus: A flexible framework for numerical simulations with spectral methods. *Physical Review Research* **2,** 023068 (2020).

6. Darnton, N. C., Turner, L., Rojevsky, S. & Berg, H. C. Dynamics of bacterial swarming. *Biophysical Journal* **98,** 2082–2090 (2010).

7. Harshey, R. M. Bacterial motility on a surface: many ways to a common goal. *Annual Reviews in Microbiology* **57,** 249–273 (2003).

8. Farrell, F. D. C., Hallatschek, O., Marenduzzo, D. & Waclaw, B. Mechanically Driven Growth of Quasi-Two-Dimensional Microbial Colonies. *Phys. Rev. Lett.* **111,** 168101 (2013).

9. Kawasaki, K., Mochizuki, A., Matsushita, M., Umeda, T. & Shigesada, N. Modeling spatio-temporal patterns generated bybacillus subtilis. *Journal of theoretical biology* **188,** 177–185 (1997).

10. Jeckel, H. *et al.* Learning the space-time phase diagram of bacterial swarm expansion. *Proceedings of the National Academy of Sciences* **116,** 1489–1494. ISSN: 0027-8424 (2019).

11. Adler, J. Chemotaxis in bacteria. *Annual Review of Biochemistry* **44,** 341–356 (1975).

12. Croze, O. A., Ferguson, G. P., Cates, M. E. & Poon, W. C. Migration of chemotactic bacteria in soft agar: role of gel concentration. *Biophysical Journal* **101,** 525–534 (2011).

13. Du, Q., Faber, V. & Gunzburger, M. Centroidal Voronoi tessellations: Applications and algorithms. *SIAM Review* **41,** 637–676 (1999).

14. Fritsch, R., Fritsch, R., Fritsch, G. & Fritsch, G. *Four-Color Theorem* (Springer, 1998).

15. Wilson, R. A. *Graphs, Colourings and the Four-colour Theorem* (OUP Oxford, 2002).

16. Gómez-Gálvez, P. *et al.* Scutoids are a geometrical solution to three-dimensional packing of epithelia. *Nature Communications* **9,** 1–14 (2018).

17. Grünbaum, B. & Shephard, G. C. *Tilings and Patterns* (Courier Dover Publications, 1987).

18. Zong, C. Packing, covering and tiling in two-dimensional spaces. *Expositiones Mathematicae* **32,** 297–364 (2014).

19. Aloupis, G., Pérez-Rosés, H., Pineda-Villavicencio, G., Taslakian, P. & Trinchet-Almaguer, D. Fitting voronoi diagrams to planar tesselations. *International Workshop on Combinatorial Algorithms,* 349–361 (2013).

20. Banerjee, S. *et al.* On the Construction of a Generalized Voronoi Inverse of a Rectangular Tessellation. *2012 Ninth International Symposium on Voronoi Diagrams in Science and Engineering,* 132–137 (2012).

21. Ash, P. F. & Bolker, E. D. Recognizing Dirichlet tessellations. *Geometriae Dedicata* **19,** 175–206. ISSN: 0046-5755 (1985).

22. Schoenberg, F. P., Ferguson, T. & Li, C. Inverting Dirichlet Tessellations. *The Computer Journal* **46,** 76–83. ISSN: 0010-4620 (2003).

23. Biedl, T., Held, M. & Huber, S. Recognizing Straight Skeletons and Voronoi Diagrams and Reconstructing Their Input. *2013 10th International Symposium on Voronoi Diagrams in Science and Engineering,* 37–46 (2013).

24. Robertson, N., Sanders, D., Seymour, P. & Thomas, R. A new proof of the four-colour theorem. *Electronic Research Announcements of the American Mathematical Society* **2,** 17–25 (1996).

25. Kylilis, N. *et al.* Whole-cell biosensor with tunable limit of detection enables low-cost agglutination assays for medical diagnostic applications. *ACS Sensors* **4,** 370–378 (2019).

26. Hicks, M., Bachmann, T. T. & Wang, B. Synthetic biology enables programmable cell-based biosensors. *ChemPhysChem* **21,** 132–144 (2020).

27. Carbonell-Ballestero, M., Garcıa-Ramallo, E., Montañez, R., Rodrıguez-Caso, C. & Macıa, J. Dealing with the genetic load in bacterial synthetic biology circuits: convergences with the Ohm's law. *Nucleic Acids Research* **44,** 496–507 (2016).

28. Liu, C. *et al.* Sequential establishment of stripe patterns in an expanding cell population. *Science* **334,** 238–241 (2011).

29. Mitchell, L. S. & Colwell, L. J. Comparative analysis of nanobody sequence and structure data. *Proteins: Structure, Function, and Bioinformatics* **86,** 697–706 (2018).

30. Olichon, A. & Marco, A. d. in *Single Domain Antibodies* 65–78 (Springer, 2012).

31. Greenwald, N. F. *et al.* Whole-cell segmentation of tissue images with human-level performance using large-scale data annotation and deep learning. *bioRxiv* (2021).

32. Zhang, R., Turner, L. & Berg, H. C. The upper surface of an Escherichia coli swarm is stationary. *Proceedings of the National Academy of Sciences* **107,** 288–290 (2010).

33. Fan, H. & Guo, Z. Bioinspired surfaces with wettability: biomolecule adhesion behaviors. *Biomaterials Science* **8,** 1502–1535 (2020).
